# Changes in Susceptibility to Heat During the Summer: A Multicountry Analysis

**DOI:** 10.1093/aje/kwv260

**Published:** 2016-05-02

**Authors:** Antonio Gasparrini, Yuming Guo, Masahiro Hashizume, Eric Lavigne, Aurelio Tobias, Antonella Zanobetti, Joel D. Schwartz, Michela Leone, Paola Michelozzi, Haidong Kan, Shilu Tong, Yasushi Honda, Ho Kim, Ben G. Armstrong

**Keywords:** adaptation, climate change, distributed lag models, heat, mortality, temperature

## Abstract

Few studies have examined the variation in mortality risk associated with heat during the summer. Here, we apply flexible statistical models to investigate the issue by using a large multicountry data set. We collected daily time-series data of temperature and mortality from 305 locations in 9 countries, in the period 1985–2012. We first estimated the heat-mortality relationship in each location with time-varying distributed lag non-linear models, using a bivariate spline to model the exposure-lag-response over lag 0–10. Estimates were then pooled by country through multivariate meta-analysis. Results provide strong evidence of a reduction in risk over the season. Relative risks for the 99th percentile versus the minimum mortality temperature were in the range of 1.15–2.03 in early summer. In late summer, the excess was substantially reduced or abated, with relative risks in the range of 0.97–1.41 and indications of wider comfort ranges and higher minimum mortality temperatures. The attenuation is mainly due to shorter lag periods in late summer. In conclusion, this multicountry analysis suggests a reduction of heat-related mortality risk over the summer, which can be attributed to several factors, such as true acclimatization, adaptive behaviors, or harvesting effects. These findings may have implications on public health policies and climate change health impact projections.

The association between high outdoor temperature and increased risk of morbidity and mortality for a number of health outcomes is conclusively established (1–4). However, important aspects remain unclear, and further evidence is needed for developing cost-effective public health policies and for providing accurate impact projections under climate change (5, 6).

In particular, researchers are currently assessing the extent of geographical and temporal variations in risks. Several studies have evaluated the excess mortality due to either heat or cold in multicity studies, revealing a considerable geographical heterogeneity across populations living in different areas (7–9). Other investigations have assessed changes in temperature-mortality associations over time, in particular examining long-term variations, and have provided evidence of an attenuation of heat-related mortality risks in several populations during the last decades (10–14). We have recently contributed to these research topics with published analyses on a large data set, assembled within the Multi-Country Multi-City collaborative network (15–17). These investigations take advantage of the application of consistent modeling approaches, based on state-of-the-art methodologies, to data from hundreds of locations around the globe, whose populations are characterized by different climatological, socioeconomic, demographic, and infrastructural features.

Changes in the susceptibility of a population to heat may also occur at shorter timescales, for example, within seasons. This issue is of interest in climate change research, as this short-term component of temporal variation can be associated with different adaptive factors if compared with the long-term changes mentioned above. For instance, a few epidemiologic studies have assessed variations in risk occurring during the summer period (7, 12, 18–20). However, these investigations were restricted to the analysis of heat-wave days (18, 19), or they approximated the complex nonlinear and delayed association between temperature and mortality through relatively simple modeling approaches (7, 12, 20). In addition, these assessments were limited to data from single cities or countries, with results difficult to compare because of different statistical methods and definitions of effect summaries.

In this contribution, we extend the research on this topic by assessing within-summer variations in heat-mortality associations in the Multi-Country Multi-City data set, including 305 locations from 9 countries and adopting flexible analytical tools based on time-varying distributed lag non-linear models (DLNMs).

## METHODS

The Multi-Country Multi-City data set has been described in a number of publications (15–17). The analytical approach adopted here is a modification of the 2-stage time-series analysis with time-varying DLNMs previously proposed (17). Details are provided below.

### The Multi-Country Multi-City data set

**Figure 1.**
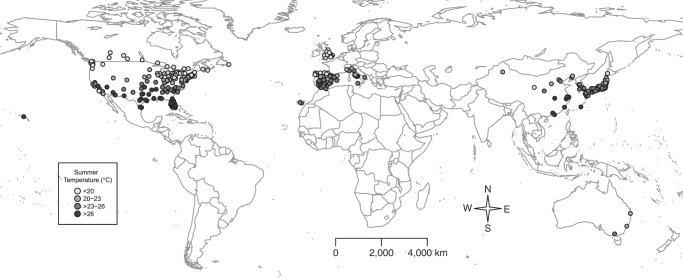
Geographical distributions of the 305 locations within the 9 countries included in the analysis, as well as the corresponding average mean daily temperature (°C) during the summer, during different study periods from 1985 to 2012.

We used a subset of the data set including data from 305 locations in 9 countries: Australia (3 cities in the period 1988–2009); Canada (26 cities, 1986–2011); China (15 cities, 2004–2008); Italy (11 cities, 1995–2006); Japan (47 prefectures, 1985–2012); South Korea (7 cities, 1992–2010); Spain (51 cities, 1990–2010); the United Kingdom (10 regions, 1990–2012); and the United States (135 cities, 1985–2009). The data consisted of daily time series of mortality counts and weather variables. Specifically, mortality was represented by daily counts of deaths for all causes or, if not available, for nonexternal causes only (*International Classification of Diseases, Ninth Revision*, codes 0–799; *International Classification of Diseases, Tenth Revision,* codes A00–R99). “Daily temperature” was defined as the 24-hour average computed from a single monitor close to the major metropolitan area or by pooling measurements from multiple monitoring stations within each location. We restricted the data to the summer period, identified as the 4 warmest months of the year using average monthly temperatures. These months consistently corresponded to the period December-March in Australia and to June-September in the other countries. Figure 1 illustrates the geographical distribution of the 305 locations and the corresponding average summer temperatures. Web Appendix 1 (available at http://aje.oxfordjournals.org/) provides additional details on data collection and references, with the full list of locations in Web Table 1.

### First-stage regression model

We first derived estimates of location-specific temperature-mortality associations by running a time-series regression model for seasonal data, based on a generalized linear model with a quasi-Poisson family (21). In this first-stage regression, seasonality was controlled for by using natural cubic B-splines of day of the season with equally spaced knots and 4 df. An interaction between this spline function and indicators of summer/year was specified to relax the assumption of a constant seasonal trend. In addition, we controlled for long-term trends by including a natural cubic B-spline of time with equally spaced knots and approximately 1 df every 10 years. The model also included indicator variables for day of the week. In the basic model, we specified the temperature-mortality association with a DLNM (22). This class of models can describe complex nonlinear and lagged dependencies through bidimensional exposure-lag-response surfaces, obtained by the combination of 2 functions that define the conventional exposure-response relationship and the additional lag-response relationship, respectively. Specifically, we selected quadratic B-splines for the exposure-response with 1 internal knot placed at the 75th percentile of location-specific summer temperature distributions and natural cubic B-splines for the lag-response with an intercept and 2 internal knots placed at equally spaced values in the log scale. The lag period was extended to 10 days in order to capture the delay in the heat-mortality association and to account for short-term harvesting. These 2 functions, with 3 and 4 df each, respectively, were combined through a cross-basis parameterization, defining 12 cross-basis variables with associated parameters that represent the whole exposure-lag-response surface, expressed as relative risk (22).

### Time-varying DLNM

The DLNMs described above assume that the bidimensional exposure-lag-response associations between high temperature and mortality, estimated in each location, are constant in time. We relaxed this assumption by extending the framework to time-varying DLNMs, defined through linear interactions between day of the season and the cross-basis variables. This extension allowed the association between temperature and mortality to change during the summer. In order to simplify the interpretation and make use of the standard DLNM software, we derived a special parameterization by directly defining the main and interaction terms in the model. The former were represented by the cross-basis variables described above, while the latter were produced by multiplying the main terms with the variable for day of the season centered at alternative values. In this parameterization, the main terms represented the exposure-lag-response dependency for the day corresponding to the centering point of the time variable. Using this time-varying DLNM, we estimated the coefficients representing the exposure-lag-response association for early and late summer, by centering on the days corresponding to the midpoint of the first and last summer months.

In order to reduce the dimensionality of the second-stage model, while at the same time preserving the complexity of the estimated dependency, we reduced the 12 coefficients of the cross-basis to unidimensional summaries (23). Specifically, we derived sets of 3 and 4 coefficients of B-splines that model the overall cumulative exposure-response relationship and the lag-response relationship at the 99th temperature percentiles, respectively. This reduction was applied to the coefficients of models with both standard and time-varying DLNMs. The former assumes no change in time, and therefore its estimates can be interpreted as an average temperature-mortality association. The latter can be used to obtain the predicted association for early and late summer, as defined above.

The first-stage regression was performed with the R software, version 3.2.0 (R Foundation for Statistical Computing, Vienna, Austria) (24), using functions in the package *dlnm*, version 2.1.4 (25).

### Second-stage meta-analysis

We separately pooled the sets of 3 and 4 parameters representing the overall cumulative exposure-response and the lag-response relationships from each model in multivariate random-effects meta-regression (23, 26). This model included indicators for country, allowing average country-specific temperature-mortality relationships. In order to partially account for the residual between-location heterogeneity attributed to different temperature distributions within countries, we also included average and range of daily temperature in the series from each location as additional meta-predictors. In addition, we derived location-specific estimates as the empirical best linear unbiased prediction, which represents a trade-off between the location-specific relationship provided by the first-stage regression and the pooled relationship (26, 27).

The second-stage meta-analysis was performed with the R package *mvmeta*, version 0.4.7 (26).

### Prediction and significance tests

From the meta-regression model, we derived country-specific predictions by setting the other meta-variables, average temperature and temperature range, to the average of each country. The predicted parameters, with associated (co)variance matrices, can be interpreted as country-pooled coefficients and used to reconstruct country-specific relationships. Consistently with the approach adopted in the first-stage regression, with knots placed at location-specific percentiles, we represented the curves on a relative scale, along percentiles of the country-specific average summer temperature distribution. Location-specific overall cumulative exposure-response curves, represented in the original temperature range for each location, were based on best linear unbiased predictions.

An assessment of the within-summer variation in the risk associated with high temperature was obtained by the comparison of the estimated curves in early and late summer. To ease interpretation, we scaled these curves by recentering them on the minimum mortality percentile of the summer temperature distribution of each country. The minimum mortality percentile was defined as the minimum of the exposure-response curve from the model with no interaction, and it was interpreted as the average optimal summer temperature corresponding to the minimum mortality risk. We also summarized the results by computing the relative risk at the 90th and 99th percentiles from these curves, using the minimum mortality percentile as the reference. Country-pooled lag-response relationships at the 99th temperature percentile were also derived from the recentered estimates by using location-specific minimum mortality percentiles derived from best linear unbiased predictions.

The statistical evidence for the difference between curves for early and late summer was assessed more formally through significance tests. We defined such tests by treating the location-specific interaction terms as cross-basis variables, reducing their coefficients and finally pooling them by country, similarly to the main terms. These sets of coefficients represented the change in the overall cumulative exposure-response curves in each country. We undertook a multivariate Wald test on these coefficients predicted in each country, accounting for the associated (co)variance matrix and assuming multivariate normality. The null hypothesis of the test was that none of the coefficients are different from 0, and therefore there was no change in the overall cumulative exposure-response association throughout the summer.

### Sensitivity analysis

In order to test the sensitivity of the results to the modeling choices described above, we repeated the analysis by varying the flexibility of the exposure-response function, using quadratic B-splines with no knots (2 df) or with 2 knots at the 50th and 90th percentiles (4 df). In addition, we assessed the sensitivity to controlling for relative humidity (with natural cubic splines with 3 df of the average over lag 0–1) and diurnal temperature range (as a liner term) in the first-stage regression, by repeating the analysis in the subset of locations for which such measures were available in at least 80% of the days (223 and 273 locations, respectively).

An updated version of the R code and data to reproduce the analysis for the United Kingdom is available in a single zipped file available on the personal web page of the first author (www.ag-myresearch.com). The R code for the analysis on the full data is available on request. We provide details in Web Appendix 2.

## RESULTS

Descriptive statistics of mortality and temperature distribution are displayed in Table 1. The data set includes 22,321,155 deaths (for all causes or nonaccidental causes only) occurring during the summer within the study periods in the 305 locations of the 9 countries. The table also compares the distribution of summer daily mean temperature between locations within each country, in early summer (first 2 months) and late summer (last 2 months). The temperature distributions appear similar.

**Table 1.** Descriptive Statistics by Country, Including the Average Mean Daily Temperature Distribution (°C) in Early (First 2 Summer Months) and Late (Last 2 Summer Months) Summer. During Different Study Periods From 1985 to 2012

Country and Summer Period	No. of Locations	Total Deaths	Study Period	Summer Temperature, °C				
				Minimum	25th Percentile	Median	75th Percentile	Maximum
Australia	3	361,135^a^	1988–2009					
Early				14.5	20.2	22.0	24.1	32.3
Late				14.3	20.1	21.7	23.7	32.1
Canada	26	944,105^b^	1986–2011					
Early				6.6	15.8	18.2	20.6	28.9
Late				3.8	14.0	16.9	19.5	28.2
China	15	291,575^a^	2004–2008					
Early				17.0	23.8	26.0	27.8	32.4
Late				15.0	21.8	24.4	26.5	31.7
Italy	11	249,828^a^	1995–2006					
Early				12.6	21.8	23.9	25.9	31.2
Late				12.5	20.0	22.6	25.0	32.0
Japan	47	8,117,084^b^	1985–2012					
Early				15.1	21.5	23.7	26.2	31.5
Late				15.3	23.2	25.6	27.5	31.8
South Korea	7	548,295^b^	1992–2010					
Early				15.3	21.6	23.4	25.5	31.5
Late				14.8	21.5	24.0	26.4	31.1
Spain	51	1,053,502^b^	1990–2010					
Early				13.0	20.2	22.5	24.6	31.5
Late				12.7	20.1	22.3	24.4	31.4
United Kingdom	10	3,654,558^b^	1990–2012					
Early				7.6	13.9	15.5	17.3	24.0
Late				8.2	13.9	15.4	17.1	25.4
United States	135	7,101,073^a^	1985–2006					
Early				13.9	22.1	24.1	26.0	32.1
Late				11.1	20.8	23.2	25.2	31.6

^a^ Deaths for nonaccidental causes only.

^b^ Deaths for all causes.

**Figure 2.**
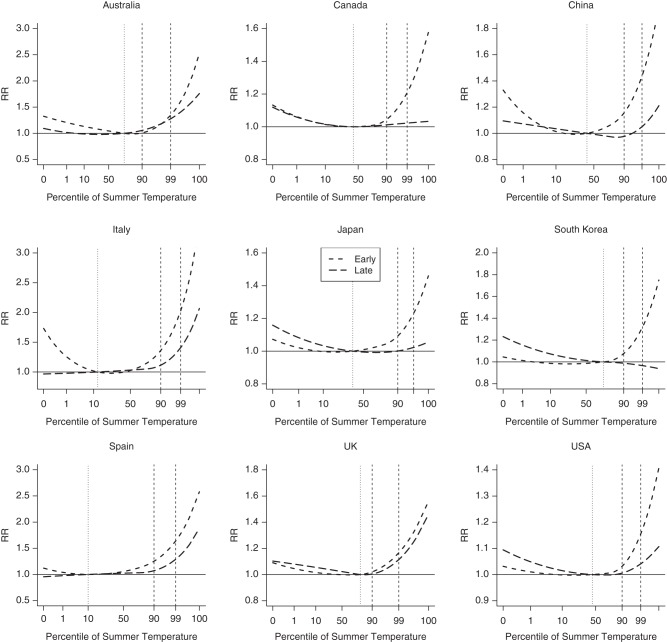
Overall cumulative exposure-response relationships between heat and mortality predicted for early (corresponding to the midpoint of the first summer month) and late (corresponding to the midpoint of the last summer month) summer in 9 countries during different study periods from 1985 to 2012. The curves are represented on a relative scale of summer temperature percentiles, using country-specific distributions. The vertical lines represent the average percentile of minimum mortality temperature (dotted) and the 90th and 99th percentiles of the temperature distribution (dashed). Note that the *y*-axis is scaled to the country-specific range. The corresponding graphs with colors and confidence intervals are added in Web Figure 1. RR, relative risk; UK, United Kingdom; USA, United States.

The main results of the analysis are summarized in Figure 2 and Table 2. Figure 2 displays the comparison of country-pooled, overall-cumulative exposure-response curves estimated from the time-varying DLNM for early and late summer, corresponding to the middle of the first and last summer months, respectively. Note that the *y* axis is scaled to the country-specific ranges, to highlight within-country differences. Table 2 reports the overall cumulative relative risk at the 90th and 99th percentiles predicted for the 2 periods and for the average across the summer, with the latter computed from the model without interaction. Plots in colors and including confidence intervals are displayed in Web Figure 1, while curves for the average across the summer and for location-specific estimates are shown in Web Figures 2 and 3, respectively. These results suggest that susceptibility to heat decreases during the summer in several countries, with the excess risk associated with heat decreasing substantially in late summer when compared with earlier in the season. Across the 9 countries, the relative risk for the 99th percentile versus the minimum mortality percentile decreases from 1.15–2.03 in early summer to 0.97–1.41 in late summer. In addition, as the season progresses, we note an increase of the minimum mortality percentiles (China, Japan, United States) and/or an extension of the range of temperatures associated with no or low risk (Canada, China, Italy, Spain). These changes suggest an upward shift in the optimal temperature and the extension of the comfort range during the summer. A more formal assessment of the evidence of an attenuation across the summer is provided by the significance tests of interaction across the whole temperature range, reported in Table 2, with the *P* values consistently indicating significant changes in China, Italy, Japan, South Korea, Spain, and the United States. Additional information is provided in Web Figure 4, illustrating the country-pooled curves of the interaction terms, interpretable of the late/early ratio of relative risks at each temperature. In particular, the significant decrease at high temperature corresponds to the reduction of relative risk in late summer. Figure 2 shown a similar decrease in Canada, with the excess risk being substantially attenuated, although the statistical evidence from the overall test of interaction is less strong. Results for Australia and the United Kingdom are too imprecise to draw conclusions, although a similar pattern can be detected.

**Table 2.** Results by Country, Including the Summer Period Used for Prediction as Average, in Early (Corresponding to the Midpoint of the First Summer Month) and Late (Corresponding to the Midpoint of the Last Summer Month) Summer, in Different Study Periods From 1985 to 2012

Country and Summer Period		Relative Risk for Mortality				*P* Value^a^
	MMP^b^	90th vs. MMP		99th vs. MMP		
		Estimated	95% CI	Estimated	95% CI	
Australia						
Whole	73rd	1.04	1.01, 1.06	1.30	1.17, 1.45	0.023
Early		1.01	0.97, 1.05	1.34	1.10, 1.64	
Late		1.06	1.02, 1.09	1.28	1.08, 1.51	
Canada						
Whole	42nd	1.03	1.01, 1.05	1.14	1.09, 1.19	0.116
Early		1.05	1.01, 1.08	1.21	1.10, 1.33	
Late		1.01	0.98, 1.04	1.02	0.93, 1.12	
China						
Whole	41st	1.06	1.02, 1.09	1.27	1.19, 1.36	0.001
Early		1.16	1.08, 1.24	1.44	1.21, 1.71	
Late		0.98	0.92, 1.03	1.05	0.90, 1.22	
Italy						
Whole	13th	1.26	1.21, 1.32	1.85	1.69, 2.02	<0.001
Early		1.36	1.26, 1.47	2.03	1.70, 2.43	
Late		1.11	1.04, 1.20	1.41	1.20, 1.65	
Japan						
Whole	38th	1.04	1.03, 1.05	1.10	1.07, 1.13	<0.001
Early		1.09	1.07, 1.11	1.23	1.16, 1.30	
Late		1.00	0.99, 1.01	1.02	0.99, 1.06	
South Korea						
Whole	71st	1.02	1.00, 1.04	1.11	1.02, 1.20	0.009
Early		1.08	1.03, 1.12	1.32	1.09, 1.59	
Late		0.99	0.96, 1.02	0.97	0.85, 1.10	
Spain						
Whole	10th	1.16	1.14, 1.18	1.45	1.40, 1.50	<0.001
Early		1.25	1.21, 1.30	1.64	1.50, 1.79	
Late		1.07	1.03, 1.11	1.29	1.20, 1.39	
United Kingdom						
Whole	79th	1.01	1.01, 1.02	1.14	1.09, 1.19	0.005
Early		1.02	1.01, 1.03	1.17	1.07, 1.27	
Late		1.00	1.00, 1.01	1.11	1.05, 1.18	
United States						
Whole	44th	1.02	1.01, 1.03	1.09	1.07, 1.11	<0.001
Early		1.03	1.02, 1.04	1.15	1.11, 1.20	
Late		1.01	1.00, 1.02	1.04	1.01, 1.07	

Abbreviations: CI, confidence interval; DLNM, distributed lag non-linear model; MMP, minimum mortality percentile.

^a^ Significance test on temporal variation, based on a multivariate Wald test of the pooled reduced coefficients of the interaction terms. The null hypothesis is that no change during the season occurred.

^b^ Estimated as the minimum of the overall cumulative exposure-response curve from the DLNM without interaction (interpreted as the average across the whole summer period).

**Figure 3.**
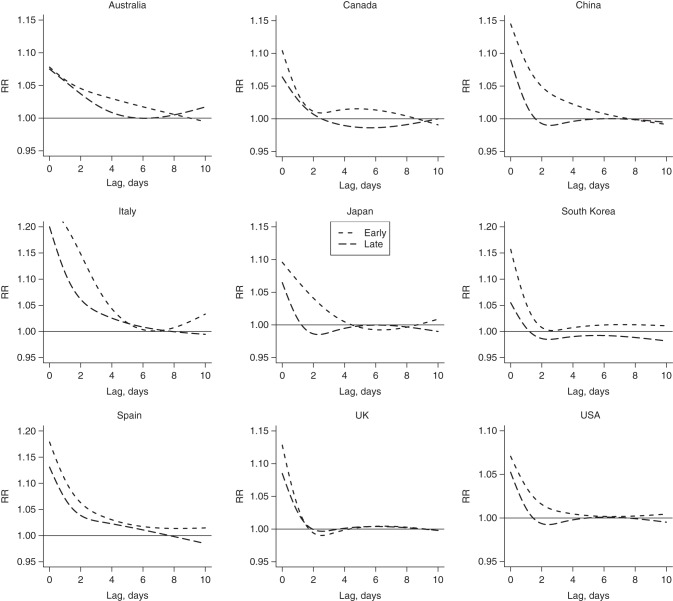
Lag-response relationships between heat and mortality predicted for early (corresponding to the midpoint of the first summer month) and late (corresponding to the midpoint of the last summer month) summer in 9 countries during different study periods from 1985 to 2012. These curves are computed for the temperature corresponding to the 99th percentile versus the average location-specific minimum mortality temperature. Note that the *y*-axis is scaled to the country-specific range. The corresponding graphs with colors and confidence intervals are added in Web Figure 5. RR, relative risk; UK, United Kingdom; USA, United States.

Figure 3 displays the lag-response associations at the 99th summer temperature percentile for early and late summer, computed versus location-specific minimum mortality percentiles, pooled by country. The corresponding relationships including confidence intervals and as the average throughout the summer are provided in Web Figures 5 and 6. The comparison of the curves suggests that in most countries the higher relative risk in early summer is largely due to the longer lag in the association. In early summer, the excess risk generally persists for some time after a day with high temperature, from approximately 4–5 days in Italy, Japan, and the United States to 8–10 days in Australia, Canada, China, and Spain. In contrast, significant relative risks are limited to lag 0–2 or 0–4 in late summer. The curves also show some evidence of harvesting, although these findings should be interpreted with caution, because of the uncertainty reflected in the confidence intervals.

The findings were confirmed by repeating the analysis using alternative DLNM specification and with control for potential confounders. In particular, models using 2 and 4 df for the exposure-response function estimated a similar decrease in the association with heat across the countries (Web Figures 7 and 8), while relative humidity and diurnal temperature range did not show any confounding effect (Web Figures 9 and 10).

## DISCUSSION

This epidemiologic investigation provides evidence of within-summer changes in susceptibility to high temperature in most of the countries included in the study. The analysis indicates that the estimated association is substantially reduced in late summer, with an increased optimal temperature and a wider comfort range, showing a null or lower increase in risk than earlier in the season. The higher risk in early summer seems to be largely explained by a more extended lag period, with increased risks up to 4–8 days, while the excess seems mostly limited to the first 2 days during late summer.

Our results are largely consistent with findings from previous studies examining changes in the mortality risk associated with heat waves during the summer (18, 19). The comparison with other investigations assessing continuous, nonlinear heat-mortality relationships is more complex, because of differences in modeling approaches and effect summary definitions. A study in 15 European cities in the period 1990–2000, using a double threshold model over lag 0–3, consistently reported an attenuation in the heat-mortality relationship during the course of the summer (7). An analysis for Seoul, South Korea, in the period 1993–2009, using a linear threshold model over lag 0–1, reported instead an increase in the relative risks associated with a 1°C increase, from 1.05 in early summer to 1.10 in late summer (12). However, the thresholds over which the risk increased linearly changed as well, from 22.8°C to 27.9°C, and it is therefore difficult to compare the overall association in the 2 periods. A larger study, including 148 cities in the United States in the period 1973–2006, estimated nonlinear temperature-mortality relationships suggesting a decrease in risk in mid-summer followed by an increase in late summer (20). However, the within-summer comparison is biased by the use of different reference temperatures in different months and potentially by controlling for temperature at lag 1–5 when reporting associations at lag 0. These issues highlight the advantages of our modeling technique, where results are reported by using minimum mortality temperatures estimated by the data while allowing nonlinear and delayed relationships.

The within-summer variation in mortality risk associated with heat may be related to both direct and indirect mechanisms. The former can occur following physiological acclimatization or adaptive changes. Physiological modifications following exposure to heat are well known and include an increase in sweating response and sweat dilution, attenuated core and skin temperature, decreased heart rate, and plasma volume expansion (28, 29). Although little is known about the associated mortality risk, the timing appears compatible with experimental studies, which show how acclimatization is built up following 2 weeks of protracted exposure to heat and then it disappears after 3 weeks of no exposure (30, 31). Adaptive behaviors adopted progressively during the summer represent additional direct mechanisms affecting susceptibility to heat: These include the use of light clothes, increased awareness of the health consequence of exposure to heat, greater use of cooling tools, and changes in daily routine. These factors are probably different from those responsible for the long-term attenuation in risk estimated along decades, which is likely to be associated with more structural changes, such as the increase in air conditioning prevalence, better housing conditions, or improvements in the health status of the population (10–14). The decrease in the susceptibility to heat of a population may also happen indirectly as the result of mortality displacement (also known as harvesting) (32), following the depletion of a pool of frail individuals after initial exposures to heat in early summer. The comparison of lag-response relationships in Figure 3 does not show important harvesting effects within the first 10 days after an extremely hot day, with curves in early summer showing in fact more prolonged associations along lags. However, mortality displacement occurring at longer timeframes during the summer cannot be ruled out. For instance, previous studies have provided evidence of weaker associations with heat in summers following winters with high mortality, suggesting the existence of mortality displacement at extended timescales (33–36).

Our study benefits from the possibility of comparing estimates from a large multicountry, multicity data set, derived following consistent procedures and definitions and obtained through the application of advanced statistical methods. The multicountry, multicity data represent the largest set of time-series data ever assembled for investigating temperature-mortality associations: The fact that we obtained similar results across populations exposed to different climates and characterized by different socioeconomic, demographic, and cultural features strengthens the evidence on within-summer variations in risk. The application of time-varying DLNM offers a flexible characterization of the association and the possibility to assess quantitatively the evidence through confidence intervals and significance tests. We also acknowledge some limitations. In particular, although this study provides a quantitative estimate of the decrease in susceptibility of the different populations to heat, it offers no information on the relative contribution of direct and indirect mechanisms in determining the decrease in risk. In addition, the data gathered so far within the multicountry, multicity collaboration do not include age or cause-specific mortality, preventing the performance of stratified analyses, which likely will be considered in future research.

The findings of this study have important implications. If populations can adjust to high temperature in a relatively short time, either through physiological acclimatization or through adaptive behaviors, the health burden of extended periods of heat can be lower than estimated from analysis of short-term temperature-mortality associations. Similarly, if the attenuation in risk instead occurs through indirect mechanisms such as mortality displacement, the overall burden in the long term would be lower than expected. The arguments discussed above emphasize the complexity of the physiological and temporal patterns characterizing the dependency between mortality risk and high temperature. Although the current methodological tools are not sophisticated enough to distinguish such complex phenomena, much can be learned from the application of different designs and modeling approaches, such as those described here. A more detailed picture of the complex association between high temperature and health, and of potential adaptive factors, will contribute to an accurate evaluation of the consequences of climate change and to the development of appropriate public health strategies implemented at the individual or population level.

## Supplementary Material

Web Material
