# Supplementary material for: Changes in Susceptibility to Heat During the Summer: A Multicountry Analysis
Source: Am J Epidemiol. 2016 May 2;183(11):1027–36. doi: 10.1093/aje/kwv260 (PMC4887574; doi:10.1093/aje/kwv260)
Supplement: Web Material [file supp_183_11_1027__index.html]

Changes in Susceptibility to Heat During the Summer: A Multicountry Analysis — Changes in Susceptibility to Heat During the Summer: A Multicountry Analysis — Web Material 

# Changes in Susceptibility to Heat During the Summer: A Multicountry Analysis

## Web Material

Web Material

- Web Material - Pdf file
