# Supplementary material for: Changes in Susceptibility to Heat During the Summer: A Multicountry Analysis
Source: Am J Epidemiol. 2016 May 2;183(11):1027–36. doi: 10.1093/aje/kwv260 (PMC4887574; doi:10.1093/aje/kwv260)
Supplement: Web Material [file supp_kwv260_kwv260supp.pdf]

Web Appendix

Changes in susceptibility to heat within the summer: a multi-country analysis

Antonio Gasparrini, Yuming Guo, Masahiro Hashizume, Eric Lavigne, Aurelio Tobias, Antonella Zanobetti, Joel D. Schwartz, Michela Leone, Paola Michelozzi, Haidong Kan, Shilu Tong, Yasushi Honda, Ho Kim, Ben G. Armstrong

Web Appendix 1

Australia

We collected data from Melbourne, Sydney and Brisbane between 1st of January 1988 and 31st of May 2009. Daily mortality, obtained from the Australian Bureau of Statistics, is represented by counts of deaths for non-external causes only (ICD-9: 0-799; ICD-10: A00-R99). Mean daily temperature (in ˚C) and relative humidity (in %), computed as the 24-hour average based on hourly measurements, were obtained from the Australian Bureau of Meteorology. We selected all available meteorological stations located within ≤30 km of each city's Central Business District (CBD) (7 stations in Brisbane, 7 stations in Melbourne and 11 stations in Sydney). We calculated the daily averages of climatic variables using all records from meteorological stations in each city. When there was a missing value (≤1.3%) for a particular meteorological station, observations recorded from the remaining weather stations were used to compute the daily average values. These data were used and described in previous publications (1-4).

Canada

We collected data from 20 census metropolitan areas (CMA) (see full list in Table S1 and in Figure S2 below) and 6 cities (Hamilton, Niagara, Oakville, Oshawa, Sarnia, Sault-Ste Marie) between 1st of January 1986 and 31st of December 2011. Daily mortality, obtained from Statistics Canada through access to the Canadian Mortality Database, is represented by counts of deaths for all causes. Mean daily temperature (in ˚C) and relative humidity (in %), computed as the 24-hour average based on hourly measurements, were obtained from Environment Canada. A single weather station was selected for each city using the airport monitoring station located closest to the CMA centre. These data (or a subset of them) were used and described in previous publications (3-6).

China

We collected data from the city of Anshan (1st of January 2004 to 31st of December 2006), Beijing (1st of January 2007 to 30th of September 2008), Fuzhou (1st of January 2004 to 31st of December 2006), Guangzhou (1st of January 2007 to 31st of December 2008), Hong Kong (1st of January 1996 to 31st of December 2002), Hangzhou (1st of January 2002 to 29th of December 2004), Lanzhou (1st of January 2004 to 31st of December 2008), Nanjing (1st of January 2007 to 31st of December 2010), Shanghai (1st of January 2001 to 31st of December 2004), Shenyang (1st of January 2005 to 31st of December 2008), Suzhu (1st of January 2005 to 31st of December 2008), Taiyuan (1st of January 2004 to 31st of December 2008), Tianjin(1st of January 2005 to 31st of December 2008), Wuhan (1st of January 2003 to 31st of December 2005), Wulumqi (1st of January 2006 to 31st of December 2007), and Xian (1st of January 2004 to 31st of December 2008). Daily mortality is represented by counts of deaths for non-external causes only (ICD-9: 0-799; ICD-10: A00-R99). Mean daily temperature (in ˚C) and relative humidity (in %) were computed as the 24-hour average based on hourly measurements. Measures of nitrogen dioxide (NO_2, in μg/m^3), particles (PM10, in in μg/m^3) and sulphur dioxide (SO2, in in μg/m^3) were available in the same period. Daily level of pollutants were computed as the 24-hour mean based on hourly measurements. In total,

missing data amount for 0.54% and 0.33% of the mortality and temperature series, respectively. These data were used and described in previous publications (3).

Italy

We collected data from the city of Bari (1st of January 1996 to 31st of December 2007), Bologna (1st of January 1996 to 31st of December 2010), Brescia (1st of January 1993 to 31st of December 2003), Civitavecchia (1st of January 1996 to 31st of December 2006), Frosinone (1st of January 1995 to 31st of December 2006), Genova (1st of January 1999 to 31st of December 2007), Latina (1st of January 1995 to 31st of December 2006), Palermo (1st of January 1997 to 31st of December 2001), Roma (1st of January 1987 to 31st of December 2010), Torino (1st of January 1991 an to 1st of December 1999), and Viterbo (1st of January 1995 to 1st of December 2006). Daily mortality, obtained from local mortality registries and from the rapid mortality surveillance system operational since 2004, is represented by counts of deaths for all causes. Mean daily temperature (in ˚C) and relative humidity (in %), computed as the 24-h average based on 6-h measurements, were obtained from the Meteorological Service of the Italian Air Force. A single weather station was selected for each city, using the airport monitoring station located closest to the city centre. In total, missing data amount for 1.95% and 3.30% of the mortality and temperature series, respectively. These data were used and described in previous publications (3, 7, 8).

Japan

We collected data from 47 prefectures (see full list in Table S1 and in Figure S2 below) between 1st of January 1985 and 31st of December 2012. Daily mortality, obtained from computerized death certificate data from the Ministry of Health, Labour and Welfare, Japan, is represented by counts of deaths for all causes and for non-external causes only (ICD-9: 0-799; ICD-10: A00-R99). Mean daily temperature (in ˚C) and relative humidity (in %), computed as the 24-hour average based on hourly measurements, were obtained from the Japan Meteorology Agency. A single weather station located within the urban area of the capital city was selected for each prefecture. These data were used and described in previous publications (3, 4).

South Korea

We collected data from 7 cities (see full list in Table S1 and in Figure S2 below) between 1st of January 1992 and 31st of December 2010. Daily mortality is represented by counts of deaths for all causes and for non-external causes only (ICD-9: 0-799; ICD-10: A00-R99). Mean daily temperature (in ˚C) and relative humidity (in %) were computed as the 24-hour average based on hourly measurements. Daily level of pollutants were computed as the 24-hour mean based on hourly measurements. These data were used and described in previous publications (3, 4).

Spain

We collected data from the 51 capital cities (see full list in Table S1 and in Figure S2 below) between 1st of January 1990 and 31st of December 2010. Daily mortality, obtained from Spain National Institute of Statistics, is represented by counts of deaths for all causes. Mean daily

temperature (in ˚C), computed as the 24-hour average based on hourly measurements, was obtained from Spain National Meteorology Agency. A single weather station, located within the urban area or at the near airport, was selected for each city. Single-day missing values were imputed as the average of the days before and after. For periods longer than two days no imputation was done. These data (or a subset of them) were used and described in previous publications (3, 4, 9).

UK

We collected data in 9 regions of England and in Wales (see full list in Table S1 and in Figure S2 below) between 1st of January 1990 and 30st of August 2012. Daily mortality, obtained from the Office of National Statistics, is represented by counts of deaths for all causes and for non-external causes only (ICD-9: 0-799; ICD-10: A00-R99). Mean daily temperature (in ˚C, computed from the 24-h average of hourly measurements) were obtained from the British Atmospheric Data Centre. An average of 29 stations contributed data to each regional series, from a minimum of 7 in London to a maximum of 44 in Wales. Missing values were imputed through interpolation between stations within the same region. A subset of these data were used and described in previous publications (3, 4, 10, 11).

USA

We collected data from 135 cities (see full list in Table S1 and in Figure S2 below) between 1st of January 1985 and 31st of December 2006. Daily mortality, obtained from the National Center for Health Statistics (NCHS), is represented by counts of deaths for non-external causes only (ICD-9: 0-799; ICD-10: A00-R99). Mean daily temperature (in ˚C, computed as the 24-hour average based on hourly measurements) and relative humidity (in %, computed from the 24-h average of hourly measurements of dew point temperature) were obtained from the National Climatic Data Center (NCDC) of the National Oceanic and Atmospheric Administration (NOAA). A single weather station was selected for each city in the land-based station data or NCDC, based on the proximity to the city's population centre. In 6 cities where multiple observations were missing from all the nearby monitors, hourly data from the Integrated Surface Database Lite of NCDC were converted in daily values. For 25 stations missing dew point data, dew point data were obtained from the nearest station with dew point data. These data were used and described in previous publications (3, 4, 12, 13).

Web Table 1. List of the 305 locations in 9 countries with study periods, total number of deaths and summer temperature (mean and range).

Location	*Country*	*Period*	*Total deaths*	*Summer temperature (˚C)*
**BRISBANE**	Australia	1988-2009	58836	24.2 (17.4 to 31.4)
**MELBOURNE**	Australia	1988-2009	140400	20.0 (10.6 to 35.0)
**SYDNEY**	Australia	1988-2009	161899	22.2 (14.0 to 31.8)
**ABBOTSFORD**	Canada	1986-2011	8572	17.0 (8.9 to 29.0)
**CALGARY**	Canada	1986-2011	42293	14.3 (-2.5 to 24.3)
**EDMONTON**	Canada	1986-2011	49262	15.3 (0.4 to 29.4)
**HALIFAX**	Canada	1986-2011	22407	16.9 (5.2 to 26.6)
**HAMILTON**	Canada	1986-2011	35884	18.9 (4.2 to 29.6)
**KINGSTON**	Canada	1986-2011	12362	18.8 (4.5 to 29.5)
**KITCHENER-WATERLOO**	Canada	1986-2011	22176	17.9 (3.6 to 29.8)
**LONDON ONTARIO**	Canada	1986-2011	30429	18.9 (4.3 to 30.7)
**MONTREAL**	Canada	1986-2009	79813	18.9 (3.3 to 29.3)
**NIAGARA**	Canada	1986-2011	30931	19.9 (6.2 to 30.5)
**OAKVILLE**	Canada	1986-2011	18543	19.4 (5.7 to 31.1)
**OSHAWA**	Canada	1986-2011	22707	18.3 (4.4 to 30.2)
**OTTAWA**	Canada	1986-2011	43017	18.7 (2.9 to 29.7)
**REGINA**	Canada	1986-2011	15797	16.3 (0.0 to 31.4)
**SARNIA**	Canada	1986-2011	8874	19.5 (6.0 to 30.7)
**SUDBURY**	Canada	1986-2011	13079	16.8 (1.2 to 28.8)
**SAINT JOHN NB**	Canada	1986-2011	13676	15.3 (4.0 to 25.7)
**ST. JOHN'S NFL**	Canada	1986-2011	17001	14.0 (1.2 to 24.3)
**SAULT STE. MARIE**	Canada	1986-2011	9327	16.4 (3.4 to 27.7)
**SASKATOON**	Canada	1986-2011	18102	15.8 (-1.4 to 32.1)
**THUNDER BAY**	Canada	1986-2011	11437	15.3 (0.9 to 27.3)
**TORONTO**	Canada	1986-2011	215163	19.6 (5.3 to 31.5)
**VICTORIA**	Canada	1986-2011	26457	15.7 (6.7 to 26.7)
**VANCOUVER**	Canada	1986-2011	103396	16.7 (9.2 to 28.4)
**WINDSOR**	Canada	1986-2011	20264	21.0 (6.0 to 31.5)
**WINNIPEG**	Canada	1986-2011	53136	17.2 (1.1 to 30.9)
**ANSHAN**	China	2004-2006	9404	24.2 (10.1 to 36.4)
**BEIJING**	China	2007-2008	24718	25.0 (12.5 to 30.7)
**FUZHOU**	China	2004-2006	5422	28.0 (20.3 to 32.2)
**GUANGZHOU**	China	2007-2008	17633	28.8 (23.7 to 33.5)
**HONG KONG**	China	1996-2002	67289	28.4 (21.1 to 33.8)
**HANGZHOU**	China	2002-2004	6370	26.9 (17.7 to 36.4)
**LANZHOU**	China	2004-2008	10552	17.6 (7.0 to 33.0)
**SHANGHAI**	China	2001-2004	51408	27.1 (19.4 to 34.0)
**SHENYANG**	China	2005-2008	30197	21.6 (10.0 to 28.0)
**SUZHU**	China	2005-2008	14563	27.0 (17.5 to 33.8)
**TAIYUAN**	China	2004-2008	13454	22.0 (9.9 to 30.5)
**TIANJIN**	China	2005-2008	4814	25.1 (12.2 to 31.3)
**WUHAN**	China	2003-2005	17896	27.6 (18.1 to 35.8)

**WULUMQI**	China	2006-2007	4048	22.1 (7.3 to 32.6)
**XIAN**	China	2004-2008	13807	23.4 (10.0 to 32.0)
**BARI**	Italy	1996-2007	9487	23.4 (11.9 to 35.2)
**BOLOGNA**	Italy	1996-2010	17787	23.8 (9.6 to 32.3)
**BRESCIA**	Italy	1993-2003	6664	22.2 (10.9 to 31.9)
**CIVITAVECCHIA**	Italy	1996-2006	1169	23.9 (16.1 to 30.3)
**FROSINONE**	Italy	1995-2006	996	23.3 (12.6 to 31.6)
**GENOA**	Italy	1999-2007	21779	23.2 (11.6 to 32.5)
**LATINA**	Italy	1995-2006	2532	23.6 (13.2 to 31.7)
**PALERMO**	Italy	1997-2001	8009	24.9 (15.2 to 37.8)
**ROME**	Italy	1987-2010	159066	23.2 (12.4 to 31.1)
**TURIN**	Italy	1991-1999	20441	20.6 (9.3 to 27.9)
**VITERBO**	Italy	1995-2006	1898	22.3 (10.6 to 32.7)
**AICHI**	Japan	1985-2012	383746	25.5 (15.5 to 32.7)
**AKITA**	Japan	1985-2012	101267	22.0 (12.1 to 31.6)
**AOMORI**	Japan	1985-2012	113976	20.4 (10.6 to 30.1)
**CHIBA**	Japan	1985-2012	313873	24.4 (13.1 to 32.2)
**EHIME**	Japan	1985-2012	116503	25.6 (16.6 to 31.9)
**FUKUSHIMA**	Japan	1985-2012	154544	22.8 (11.3 to 31.4)
**FUKUI**	Japan	1985-2012	58665	24.5 (14.6 to 32.1)
**FUKUOKA**	Japan	1985-2012	324858	25.8 (17.5 to 32.4)
**GIFU**	Japan	1985-2012	136903	25.5 (15.6 to 32.9)
**GUNMA**	Japan	1985-2012	135317	24.1 (12.6 to 32.6)
**HOKKAIDO**	Japan	1985-2012	389479	19.6 (9.8 to 30.1)
**HIROSHIMA**	Japan	1985-2012	194391	25.7 (15.6 to 32.7)
**HYOGO**	Japan	1985-2012	354299	25.7 (16.2 to 32.0)
**IBARAKI**	Japan	1985-2012	189316	22.8 (11.6 to 31.3)
**ISHIKAWA**	Japan	1985-2012	80735	24.3 (15.0 to 32.3)
**IWATE**	Japan	1985-2012	107927	20.8 (10.4 to 29.3)
**KAGAWA**	Japan	1985-2012	76721	25.8 (16.9 to 32.3)
**KANAGAWA**	Japan	1985-2012	430516	24.3 (13.1 to 30.9)
**KAGOSHIMA**	Japan	1985-2012	146568	26.7 (18.5 to 31.1)
**KOCHI**	Japan	1985-2012	70061	25.6 (15.8 to 32.1)
**KUMAMOTO**	Japan	1985-2012	134895	26.1 (16.2 to 31.5)
**KYOTO**	Japan	1985-2012	170793	25.7 (15.1 to 32.8)
**MIE**	Japan	1985-2012	127223	25.3 (15.9 to 33.5)
**MIYAGI**	Japan	1985-2012	142635	21.6 (10.8 to 31.2)
**MIYAZAKI**	Japan	1985-2012	84538	25.8 (16.7 to 32.0)
**NAGANO**	Japan	1985-2012	159353	22.6 (11.9 to 30.7)
**NAGASAKI**	Japan	1985-2012	115970	25.7 (17.4 to 32.2)
**NARA**	Japan	1985-2012	87912	24.5 (14.6 to 31.7)
**NIIGATA**	Japan	1985-2012	184269	23.7 (14.4 to 32.6)
**OITA**	Japan	1985-2012	94400	25.2 (16.2 to 31.6)
**OKAYAMA**	Japan	1985-2012	140908	25.9 (15.9 to 32.3)
**OKINAWA**	Japan	1985-2012	68982	28.1 (21.1 to 31.1)
**OSAKA**	Japan	1985-2012	526314	26.3 (15.5 to 32.9)

**SAGA**	Japan	1985-2012	65842	25.8 (16.0 to 32.2)
**SAITAMA**	Japan	1985-2012	343311	24.4 (12.9 to 33.7)
**SHIGA**	Japan	1985-2012	77718	24.6 (15.1 to 31.4)
**SHIMANE**	Japan	1985-2012	65113	24.2 (15.0 to 32.2)
**SHIZUOKA**	Japan	1985-2012	234281	24.9 (14.6 to 31.9)
**TOKUSHIMA**	Japan	1985-2012	66168	25.6 (16.9 to 32.3)
**TOCHIGI**	Japan	1985-2012	130789	23.3 (12.0 to 31.4)
**TOKYO**	Japan	1985-2012	710556	25.0 (13.2 to 33.1)
**TOTTORI**	Japan	1985-2012	48663	24.5 (14.5 to 32.3)
**TOYAMA**	Japan	1985-2012	84518	23.9 (15.1 to 33.8)
**WAKAYAMA**	Japan	1985-2012	85859	25.8 (16.9 to 31.9)
**YAMAGATA**	Japan	1985-2012	99932	22.3 (12.0 to 31.1)
**YAMAGUCHI**	Japan	1985-2012	124916	25.0 (13.9 to 31.2)
**YAMANASHI**	Japan	1985-2012	61561	24.4 (14.7 to 31.8)
**BUSAN**	South Korea	1992-2010	107590	23.3 (16.1 to 30.2)
**DAEGU**	South Korea	1992-2010	65670	24.4 (15.5 to 32.9)
**DAEJEON**	South Korea	1992-2010	32897	23.6 (13.0 to 31.8)
**GWANGJU**	South Korea	1992-2010	34231	24.2 (15.7 to 31.3)
**INCHEON**	South Korea	1992-2010	62066	23.0 (13.5 to 31.4)
**SEOUL**	South Korea	1992-2010	228164	23.7 (13.0 to 33.0)
**ULSAN**	South Korea	1992-2010	17677	23.6 (15.1 to 30.8)
**A CORUNA**	Spain	1990-2010	23135	18.9 (13.0 to 29.0)
**ALBACETE**	Spain	1990-2010	10495	23.0 (10.7 to 31.6)
**ALICANTE**	Spain	1990-2010	15634	24.7 (17.3 to 32.2)
**ALMERIA**	Spain	1990-2010	12709	25.2 (17.2 to 36.2)
**AVILA**	Spain	1990-2010	6736	19.0 (6.4 to 28.6)
**BADAJOZ**	Spain	1990-2010	11335	24.7 (15.1 to 33.9)
**BARCELONA**	Spain	1990-2010	110440	23.0 (14.2 to 30.9)
**BILBAO**	Spain	1990-2010	24210	19.7 (11.9 to 32.2)
**BURGOS**	Spain	1990-2010	13026	18.1 (6.6 to 29.8)
**CACERES**	Spain	1990-2010	6682	24.5 (13.6 to 34.1)
**CADIZ**	Spain	1990-2010	12588	23.8 (16.8 to 32.9)
**CASTELLON**	Spain	1990-2010	12557	24.4 (14.8 to 32.0)
**CEUTA**	Spain	1990-2010	3069	23.7 (14.9 to 32.8)
**CORDOBA**	Spain	1990-2010	21505	26.3 (15.7 to 36.3)
**CIUDAD REAL**	Spain	1990-2010	6933	24.7 (11.9 to 33.7)
**CUENCA**	Spain	1990-2010	5159	21.6 (9.2 to 29.8)
**GIRONA**	Spain	1990-2010	9619	22.0 (12.3 to 30.4)
**GRANADA**	Spain	1990-2010	23194	24.0 (12.8 to 32.7)
**GUADALAJARA**	Spain	1990-2010	6275	21.5 (10.0 to 29.0)
**HUELVA**	Spain	1990-2010	13142	24.5 (15.8 to 36.2)
**HUESCA**	Spain	1990-2010	4824	22.2 (11.3 to 32.0)
**JAEN**	Spain	1990-2010	11011	25.2 (13.1 to 35.3)
**LEON**	Spain	1990-2010	13663	18.2 (7.4 to 27.9)
**LLEIDA**	Spain	1990-2010	11309	23.4 (12.4 to 30.6)
**LOGRONO**	Spain	1990-2010	10081	21.4 (9.5 to 31.5)

**LUGO**	Spain	1990-2010	10413	17.4 (7.9 to 29.1)
**MADRID**	Spain	1990-2010	171802	23.6 (10.8 to 32.4)
**MALAGA**	Spain	1990-2010	35549	24.8 (16.4 to 34.2)
**MELILLA**	Spain	1990-2010	2699	24.5 (17.5 to 36.1)
**MURCIA**	Spain	1990-2010	23245	24.5 (17.5 to 36.1)
**OURENSE**	Spain	1990-2010	11763	21.5 (10.4 to 31.5)
**OVIEDO**	Spain	1990-2010	21508	18.1 (10.0 to 28.4)
**PAMPLONA**	Spain	1990-2010	17661	20.0 (9.1 to 31.6)
**PALMAS G. CANARIA**	Spain	1990-2010	26984	23.8 (19.0 to 33.4)
**PALMA MALLORCA**	Spain	1990-2010	25347	23.6 (15.3 to 32.1)
**PONTEVEDRA**	Spain	1990-2010	9707	19.6 (11.6 to 30.4)
**SALAMANCA**	Spain	1990-2010	13985	19.8 (9.6 to 29.1)
**SANTANDER**	Spain	1990-2010	17796	19.3 (12.5 to 27.8)
**TENERIFE**	Spain	1990-2010	16084	24.7 (20.0 to 34.3)
**SEGOVIA**	Spain	1990-2010	5424	20.3 (7.5 to 31.4)
**SEVILLA**	Spain	1990-2010	52635	26.9 (17.0 to 36.8)
**SORIA**	Spain	1990-2010	4055	18.7 (7.0 to 28.1)
**SAN SEBASTIAN**	Spain	1990-2010	20326	18.4 (10.9 to 30.3)
**TARRAGONA**	Spain	1990-2010	8439	25.0 (14.2 to 32.4)
**TERUEL**	Spain	1990-2010	3844	20.2 (8.8 to 27.8)
**TOLEDO**	Spain	1990-2010	9639	24.7 (11.3 to 34.0)
**VALENCIA**	Spain	1990-2010	63401	24.8 (14.3 to 33.8)
**VITORIA**	Spain	1990-2010	11721	18.0 (7.8 to 30.6)
**VALLADOLID**	Spain	1990-2010	20582	20.7 (9.6 to 30.9)
**ZAMORA**	Spain	1990-2010	6435	21.1 (10.4 to 30.8)
**ZARAGOZA**	Spain	1990-2010	43127	23.7 (13.3 to 32.9)
**EAST**	UK	1990-2012	362868	16.1 (7.5 to 25.2)
**EAST MIDLANDS**	UK	1990-2012	293288	15.5 (6.2 to 25.3)
**LONDON**	UK	1990-2012	401709	17.2 (9.2 to 29.1)
**NORTH EAST**	UK	1990-2012	197506	14.5 (6.0 to 25.0)
**NORTH WEST**	UK	1990-2012	515728	14.9 (7.5 to 23.7)
**SOUTH EAST**	UK	1990-2012	546649	16.2 (8.3 to 25.3)
**SOUTH WEST**	UK	1990-2012	374222	15.6 (8.5 to 24.8)
**WALES**	UK	1990-2012	228091	15.1 (8.1 to 24.0)
**WEST MIDLANDS**	UK	1990-2012	372152	15.4 (6.8 to 25.0)
**YORKSHIRE & HUMBER**	UK	1990-2012	362345	15.2 (7.0 to 26.1)
**AKRON, OH**	USA	1985-2006	33295	20.3 (6.4 to 30.8)
**ALBUQUERQUE, NM**	USA	1985-2006	22151	23.8 (9.7 to 32.2)
**ALLENTOWN-BETHLEHEM, PA**	USA	1985-2006	18616	21.2 (6.9 to 31.4)
**ATLANTA, GA**	USA	1985-2006	96303	25.4 (11.7 to 32.5)
**ATLANTIC CITY, NJ**	USA	1985-2006	15199	22.1 (8.1 to 32.2)
**AUSTIN, TX**	USA	1985-2006	21831	28.4 (14.2 to 35.0)
**BAKERSFIELD, CA**	USA	1985-2006	27716	26.5 (14.2 to 36.7)
**BALTIMORE, MD**	USA	1985-2006	99401	23.2 (9.7 to 32.5)
**BARNSTABLE-**	USA	1985-2006	16153	19.5 (7.8 to 30.6)

**YARMOUTH, MA**				
**BERGEN-PASSAIC, NJ**	USA	1985-2006	73212	23.4 (10.6 to 34.7)
**BIRMINGHAM, AL**	USA	1985-2006	53165	25.6 (13.1 to 32.2)
**BOSTON, MA**	USA	1985-2006	145615	20.9 (7.5 to 32.2)
**BATON ROUGE, LA**	USA	1985-2006	19473	27.2 (14.4 to 32.8)
**BROWNSVILLE, TX**	USA	1985-2006	10965	28.9 (17.5 to 33.1)
**BUFFALO, NY**	USA	1985-2006	65843	19.8 (5.8 to 30.3)
**CANTON-MASSILLON, OH**	USA	1985-2006	23766	20.3 (6.4 to 30.8)
**CHARLESTON, WV**	USA	1985-2006	15363	22.3 (8.1 to 31.7)
**CHARLOTTE, NC**	USA	1985-2006	25311	24.8 (11.7 to 32.5)
**CHATTANOOGA, TN**	USA	1985-2006	18913	25.0 (12.8 to 32.8)
**CHICAGO, IL**	USA	1985-2006	349050	21.1 (5.6 to 33.6)
**CINCINNATI, OH**	USA	1985-2006	53094	22.7 (9.2 to 33.1)
**CLEVELAND, OH**	USA	1985-2006	125455	20.7 (7.5 to 30.8)
**COLUMBIA, SC**	USA	1985-2006	23635	26.2 (13.3 to 32.8)
**COLUMBUS, OH**	USA	1985-2006	49586	22.0 (8.3 to 31.7)
**DALLAS, TX**	USA	1985-2006	81635	28.1 (12.2 to 35.8)
**DAYTONA BEACH, FL**	USA	1985-2006	33248	27.1 (21.1 to 31.7)
**DAYTON, OH**	USA	1985-2006	33728	21.7 (7.2 to 32.2)
**DENVER, CO**	USA	1985-2006	56583	20.7 (-5.0 to 30.3)
**DES MOINES, IA**	USA	1985-2006	16899	22.0 (4.7 to 32.8)
**DETROIT, MI**	USA	1985-2006	229193	21.0 (6.4 to 31.4)
**DUTCHESS COUNTY, NY**	USA	1985-2006	13143	20.2 (6.4 to 31.7)
**EL PASO, TX**	USA	1985-2006	22366	26.8 (11.4 to 36.7)
**ERIE, PA**	USA	1985-2006	16916	20.3 (7.5 to 30.0)
**FLINT, MI**	USA	1985-2006	23674	19.6 (4.7 to 30.8)
**FRESNO, CA**	USA	1985-2006	31852	26.3 (13.6 to 38.6)
**FT. LAUDERDALE, FL**	USA	1985-2006	97450	27.4 (23.1 to 30.8)
**FORT MYERS-CAPE CORAL, FL**	USA	1985-2006	27339	28.2 (22.8 to 31.4)
**FORT PIERCE-PORT ST. LUCIE, FL**	USA	1985-2006	20681	27.3 (22.8 to 30.8)
**FORT WORTH-ARLINGTON, TX**	USA	1985-2006	53579	28.0 (13.1 to 35.6)
**GALVESTON, TX**	USA	1985-2006	12921	27.1 (16.7 to 32.8)
**GARY, IN**	USA	1985-2006	28345	20.9 (5.3 to 31.7)
**GRAND RAPIDS, MI**	USA	1985-2006	24572	19.9 (4.4 to 31.7)
**GREENSBORO, NC**	USA	1985-2006	20318	23.7 (11.1 to 30.6)
**GREENVILLE, SC**	USA	1985-2006	17982	24.5 (11.7 to 32.8)
**HAMILTON, OH**	USA	1985-2006	15447	22.7 (9.2 to 33.1)
**HARRISBURG-CARLISLE, PA**	USA	1985-2006	15600	22.6 (8.1 to 32.5)
**HARTFORD, CT**	USA	1985-2006	48290	20.8 (5.8 to 31.1)
**HONOLULU, HI**	USA	1985-2006	24364	27.5 (24.2 to 30.3)
**HOUSTON, TX**	USA	1985-2006	115796	28.0 (15.3 to 33.3)
**INDIANAPOLIS, IN**	USA	1985-2006	46853	22.3 (7.8 to 32.2)
**JACKSONVILLE, FL**	USA	1985-2006	38724	27.0 (17.8 to 32.5)

**JERSEY CITY, NJ**	USA	1985-2006	31701	23.4 (10.6 to 34.7)
**KANSAS CITY, MO-KS**	USA	1985-2006	67807	23.5 (6.1 to 33.9)
**KNOXVILLE, TN**	USA	1985-2006	24834	24.1 (10.8 to 30.3)
**LAKELAND-WINTER HAVEN, FL**	USA	1985-2006	29250	27.9 (21.4 to 32.2)
**LANCASTER, PA**	USA	1985-2006	24548	21.7 (8.9 to 31.1)
**LANSING, MI**	USA	1985-2006	11583	19.4 (5.3 to 30.8)
**LAS VEGAS, NV-AZ**	USA	1985-2006	56467	30.7 (14.7 to 41.1)
**LOS ANGELES, CA**	USA	1985-2006	382554	20.4 (13.6 to 31.1)
**LOUISVILLE, KY**	USA	1985-2006	42618	24.2 (10.0 to 34.2)
**LITTLE ROCK, AR**	USA	1985-2006	19999	26.4 (13.3 to 35.3)
**LUBBOCK, TX**	USA	1985-2006	10691	25.2 (6.4 to 35.6)
**MADISON, WI**	USA	1985-2006	15106	19.7 (5.0 to 32.8)
**MCALLEN-EDINBURG-MISSION, TX**	USA	1985-2006	15090	29.9 (16.7 to 36.7)
**MELBOURNE-TITUSVILLE-PALM BAY, FL**	USA	1985-2006	27810	27.2 (21.9 to 31.9)
**MEMPHIS, TN**	USA	1985-2006	47476	26.5 (13.6 to 33.9)
**MIAMI, FL**	USA	1985-2006	119487	28.5 (23.3 to 31.4)
**MIDDLESEX, NJ**	USA	1985-2006	33829	21.8 (8.6 to 32.5)
**MILWAUKEE, WI**	USA	1985-2006	71799	20.3 (6.1 to 33.9)
**MINNEAPOLIS-ST. PAUL, MN**	USA	1985-2006	74244	20.4 (4.7 to 32.5)
**MOBILE, AL**	USA	1985-2006	22679	26.8 (15.0 to 32.8)
**MONMOUTH-OCEAN, NJ**	USA	1985-2006	73356	21.8 (8.1 to 31.9)
**MYRTLE BEACH, SC**	USA	1985-2006	9518	25.6 (14.7 to 33.3)
**NAPLES, FL**	USA	1985-2006	11336	28.0 (21.9 to 30.8)
**NASHUA, NH**	USA	1985-2006	15701	19.4 (6.1 to 30.0)
**NASHVILLE, TN**	USA	1985-2006	30097	24.9 (11.1 to 32.5)
**NASSAU-SUFFOLK, NY**	USA	1985-2006	143694	21.4 (8.6 to 32.2)
**NEWARK, NJ**	USA	1985-2006	68728	23.4 (10.6 to 34.7)
**NEWBURGH, NY**	USA	1985-2006	15476	20.6 (6.7 to 30.8)
**NEW HAVEN-MERIDEN, CT**	USA	1985-2006	48273	20.8 (5.8 to 31.1)
**NEW LONDON, CT**	USA	1985-2006	12576	20.7 (7.8 to 31.1)
**NEW YORK, NY**	USA	1985-2006	426430	23.4 (10.3 to 34.4)
**OAKLAND, CA**	USA	1985-2006	99888	18.1 (12.2 to 27.5)
**OCALA, FL**	USA	1985-2006	17933	27.2 (19.4 to 32.2)
**OKLAHOMA CITY, OK**	USA	1985-2006	36601	25.7 (7.5 to 34.7)
**OMAHA, NE**	USA	1985-2006	22024	22.3 (2.2 to 33.3)
**ORANGE COUNTY, CA**	USA	1985-2006	98795	22.4 (14.4 to 32.8)
**ORLANDO, FL**	USA	1985-2006	49272	27.8 (21.9 to 32.2)
**PENSACOLA, FL**	USA	1985-2006	16165	27.3 (16.9 to 34.2)
**PHILADELPHIA, PA-NJ**	USA	1985-2006	279833	23.5 (9.4 to 33.3)

**PHOENIX, AZ**	USA	1985-2006	117774	33.2 (18.9 to 41.4)
**PITTSBURGH, PA**	USA	1985-2006	97477	20.9 (7.8 to 30.3)
**PORTLAND, ME**	USA	1985-2006	14093	18.5 (5.0 to 29.7)
**PORTLAND, OR**	USA	1985-2006	65670	19.4 (10.6 to 29.7)
**PROVIDENCE-FALL RIVER, RI-MA**	USA	1985-2006	11215	20.9 (7.2 to 31.4)
**PUNTA GORDA, FL**	USA	1985-2006	11663	28.0 (23.1 to 32.2)
**RALEIGH, NC**	USA	1985-2006	18149	24.5 (12.5 to 32.2)
**READING, PA**	USA	1985-2006	22163	24.5 (12.5 to 32.2)
**RIVERSIDE-SAN BERNARDINO, CA**	USA	1985-2006	134247	25.1 (12.2 to 36.7)
**ROCHESTER, NY**	USA	1985-2006	39921	19.6 (5.0 to 30.3)
**ROCKFORD, IL**	USA	1985-2006	14176	20.7 (4.7 to 31.7)
**SACRAMENTO, CA**	USA	1985-2006	53242	23.1 (13.3 to 34.7)
**SAGINAW, MI**	USA	1985-2006	12524	19.6 (5.3 to 31.1)
**SALINAS, CA**	USA	1985-2006	14338	17.3 (12.2 to 25.8)
**SALT LAKE CITY, UT**	USA	1985-2006	27783	22.8 (5.6 to 32.8)
**SAN ANTONIO, TX**	USA	1985-2006	57736	28.4 (12.8 to 34.2)
**SARASOTA-BRADENTON, FL**	USA	1985-2006	46351	28.0 (20.6 to 32.8)
**SCRANTON--WILKES-BARRE--HAZLETON, PA**	USA	1985-2006	46123	20.0 (5.8 to 31.9)
**SAN DIEGO, CA**	USA	1985-2006	115049	20.9 (14.7 to 30.8)
**SEATTLE, WA**	USA	1985-2006	69921	17.5 (8.9 to 28.1)
**SAN FRANCISCO, CA**	USA	1985-2006	77042	17.6 (11.9 to 30.0)
**SHREVEPORT, LA**	USA	1985-2006	16221	27.2 (13.6 to 34.2)
**SAN JOSE, CA**	USA	1985-2006	54245	21.0 (13.9 to 32.2)
**SPOKANE, WA**	USA	1985-2006	21294	18.3 (4.4 to 30.6)
**SPRINGFIELD, MA**	USA	1985-2006	29030	20.8 (5.8 to 31.1)
**STAMFORD-NORWALK, CT**	USA	1985-2006	43691	21.5 (8.6 to 30.8)
**ST. LOUIS, MO-IL**	USA	1985-2006	96368	24.6 (9.2 to 33.6)
**STOCKTON-LODI, CA**	USA	1985-2006	25244	23.8 (13.9 to 36.9)
**SYRACUSE, NY**	USA	1985-2006	26252	19.8 (5.0 to 30.3)
**TACOMA, WA**	USA	1985-2006	29688	17.8 (10.0 to 26.7)
**TAMPA-ST. PETERSBURG-CLEARWATER, FL**	USA	1985-2006	49811	28.0 (21.7 to 31.7)
**TOLEDO, OH**	USA	1985-2006	28669	20.9 (6.9 to 31.7)
**TRENTON, NJ**	USA	1985-2006	17869	21.6 (7.8 to 31.7)
**TUCSON, AZ**	USA	1985-2006	39643	29.5 (18.6 to 37.2)
**TULSA, OK**	USA	1985-2006	29746	26.1 (8.6 to 35.3)
**UTICA-ROME, NY**	USA	1985-2006	16479	19.0 (4.4 to 28.9)
**VENTURA COUNTY, CA**	USA	1985-2006	27134	18.9 (13.1 to 26.4)
**VIRGINIA BEACH, VA**	USA	1985-2006	58165	24.9 (13.9 to 33.1)
**WASHINGTON, DC-MD-VA**	USA	1985-2006	44237	24.4 (10.6 to 33.9)
**WICHITA, KS**	USA	1985-2006	21215	24.9 (5.0 to 33.9)

**WILMINGTON, DE**	USA	1985-2006	23455	22.8 (9.2 to 31.9)
**WORCESTER, MA**	USA	1985-2006	41347	19.0 (3.3 to 28.9)
**WEST PALM BEACH-BOCA RATON, FL**	USA	1985-2006	72824	28.0 (23.3 to 31.1)
**YORK, PA**	USA	1985-2006	19280	22.0 (8.3 to 31.1)
**YOUNGSTOWN-WARREN, OH**	USA	1985-2006	26632	19.6 (6.4 to 30.6)

Web Appendix 2

The analysis of the multi-country data set was performed in R, version 3.2.1.(14) The first and second-stage models were performed using the two packages `dlnm` (version 2.1.4) and `mvmeta` (version 0.4.7), respectively (15, 16).

The R code and data reproducing all the steps of the analysis are provided in a single zipped file as Supplemental Material on the Journal's website. An update version is also available at the personal web page of the first author (http://www.ag-myresearch.com/), and at the time of writing can be accessed via the sections 'Publications' (through the link to this article) or 'R code'. The code will be updated consistently with the development of the two R packages.

The code consists of a series of scripts to be run consecutively. Although the full results cannot be reproduced as most of the data are not publicly available, we provide a subset of the dataset for the 10 regions of the UK used in the analysis. We anticipate that the results are similar yet not identical to those reported in the article, as the latter are based on the full data and a different dataset for the UK.

Web Figure 1. Replication of Figure 2 in the manuscript, including colours and confidence intervals as shaded areas.

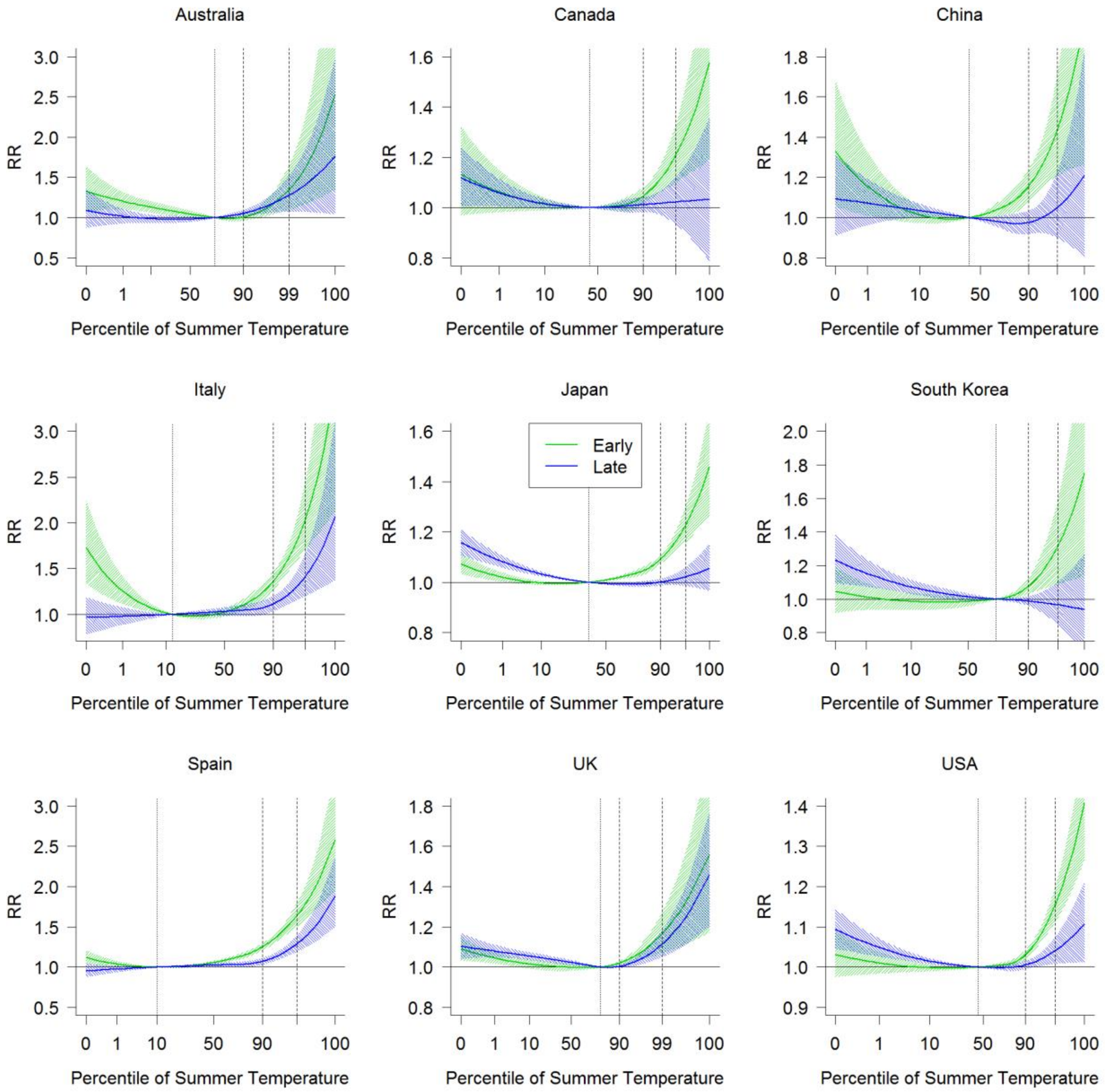

Web Figure 2. Replication of Figure 2 in the manuscript for the average across the summer, with confidence intervals as grey areas.

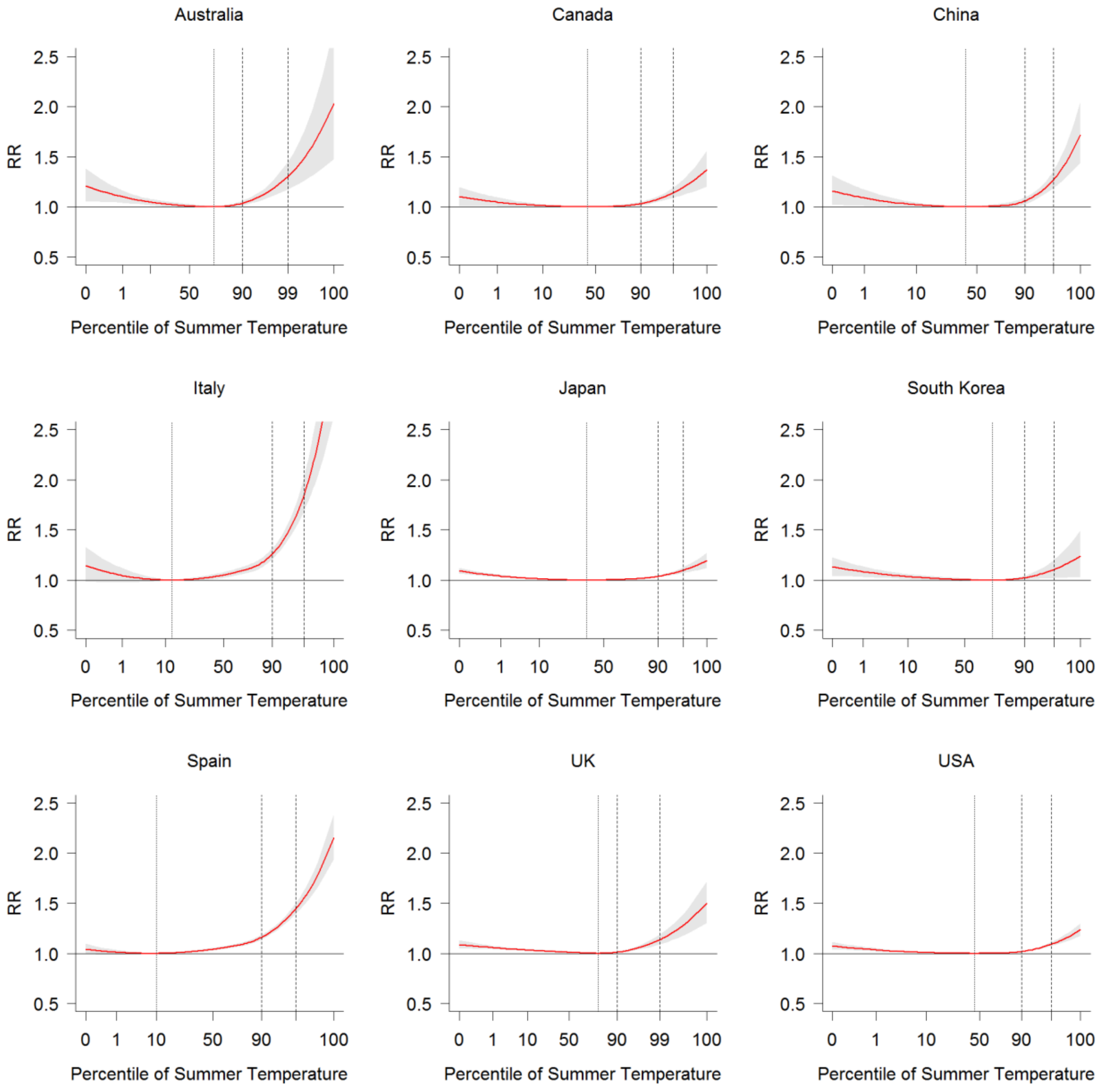

Web Figure 3. Overall cumulative exposure-response relationships[a] between heat and mortality predicted for early (corresponding to the mid-point of the first summer month) and late summer (corresponding to the mid-point of the last summer month) in 305 locations, with 95% confidence intervals. The curves are represented on a relative scale of summer temperature percentiles, using country-specific distributions. Different study periods in 1985-2012.

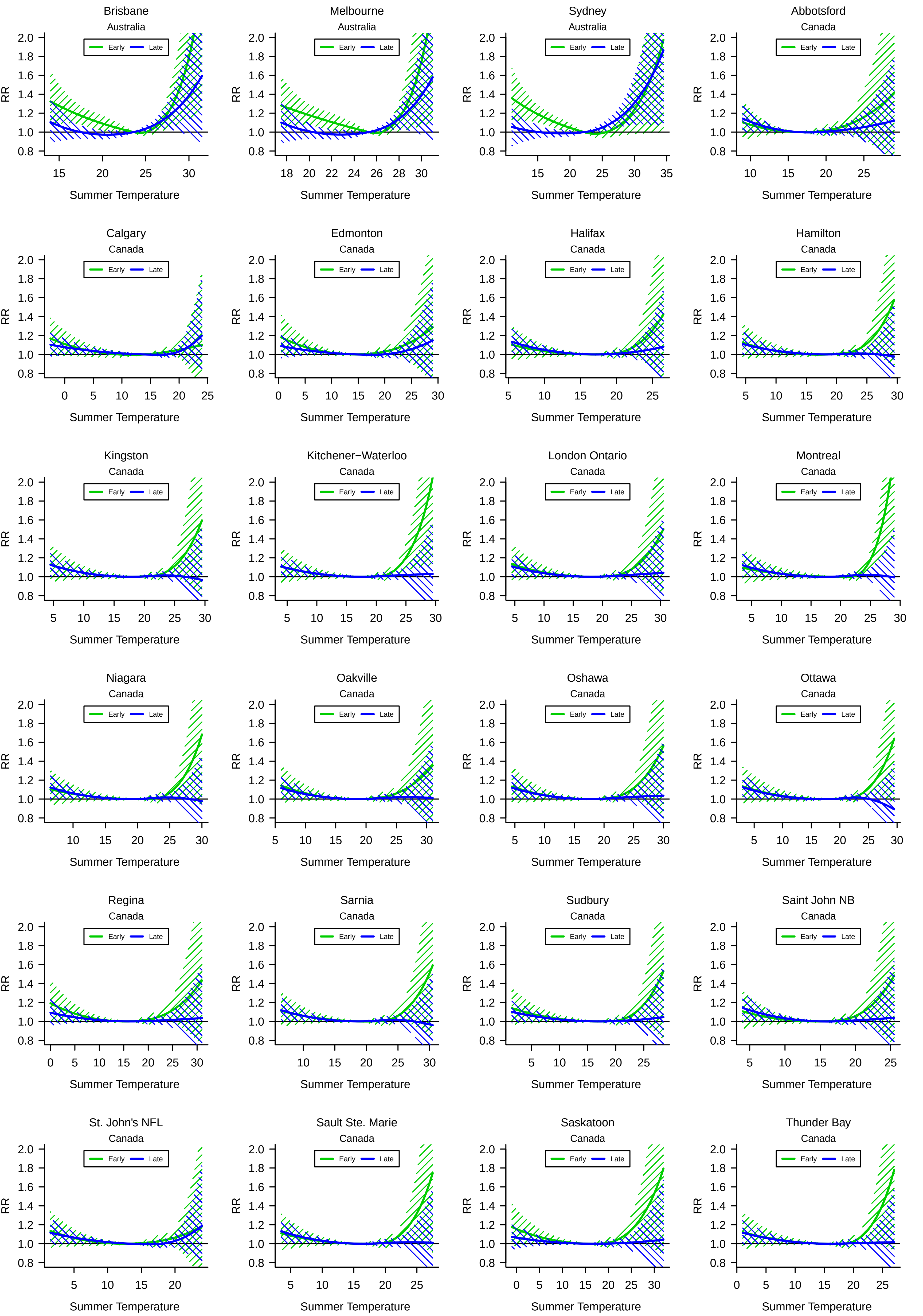

Brisbane
Australia
Melbourne
Australia
Sydney
Australia
Abbotsford
Canada
Calgary
Canada
Edmonton
Canada
Halifax
Canada
Hamilton
Canada
Kingston
Canada
Kitchener−Waterloo
Canada
London Ontario
Canada
Montreal
Canada
Niagara
Canada
Oakville
Canada
Oshawa
Canada
Ottawa
Canada
Regina
Canada
Sarnia
Canada
Sudbury
Canada
Saint John NB
Canada
St. John's NFL
Canada
Sault Ste. Marie
Canada
Saskatoon
Canada
Thunder Bay
Canada
Early
Late
RR
Summer Temperature

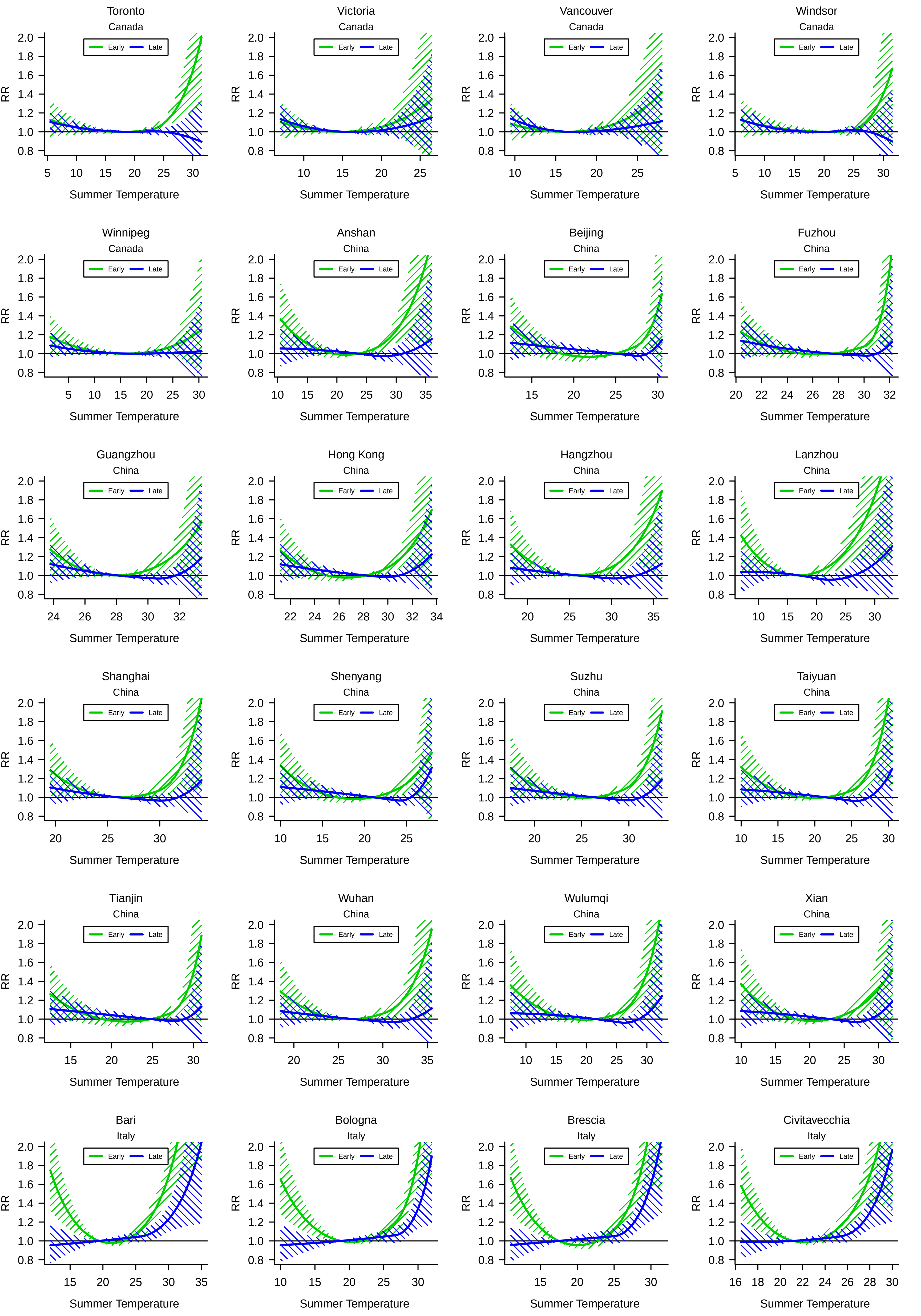
Toronto
Canada
Victoria
Canada
Vancouver
Canada
Windsor
Canada
Winnipeg
Canada
Anshan
China
Beijing
China
Fuzhou
China
Guangzhou
China
Hong Kong
China
Hangzhou
China
Lanzhou
China
Shanghai
China
Shenyang
China
Suzhu
China
Taiyuan
China
Tianjin
China
Wuhan
China
Wulumqi
China
Xian
China
Bari
Italy
Bologna
Italy
Brescia
Italy
Civitavecchia
Italy
Early
Late
RR
Summer Temperature

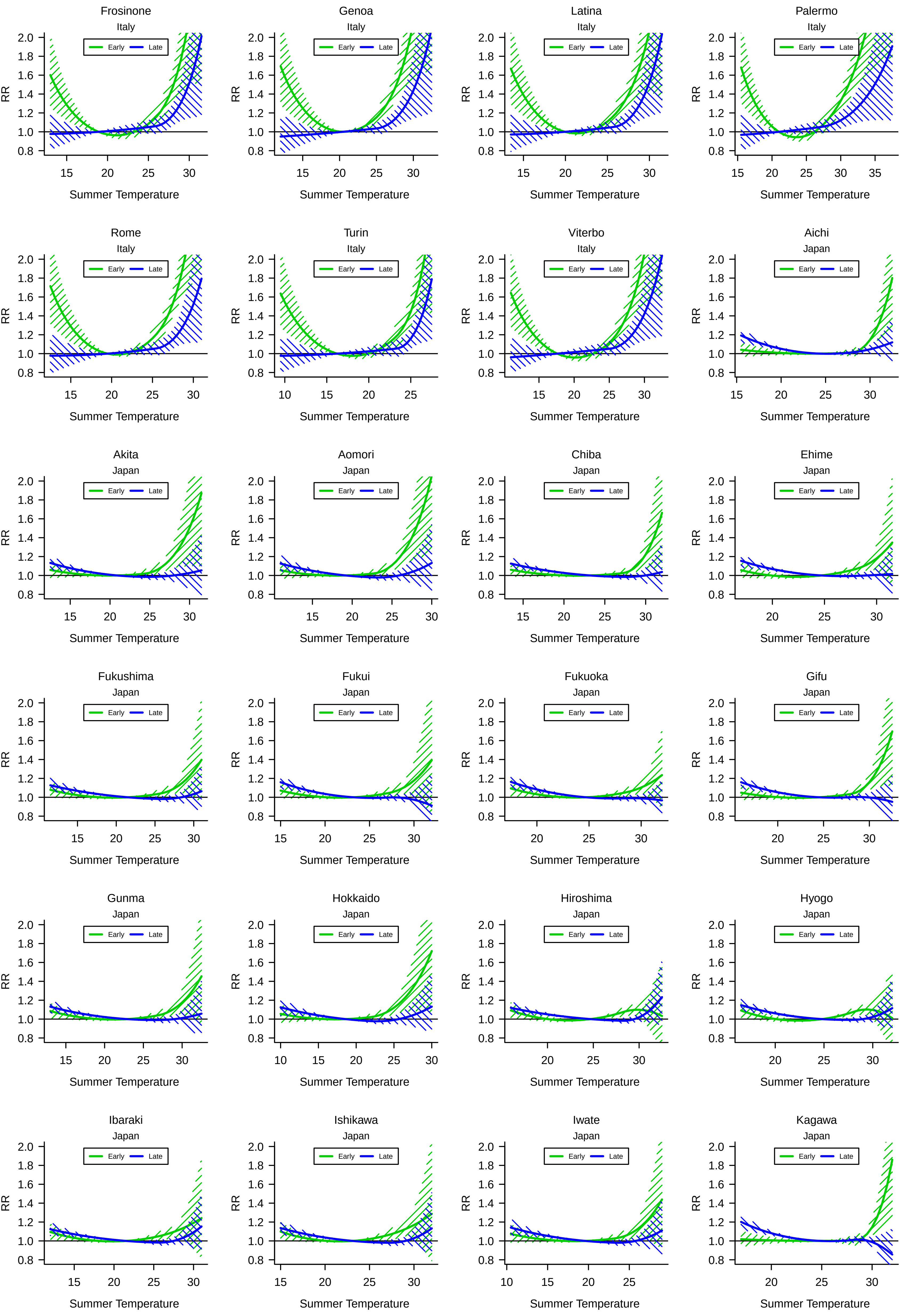
Frosinone
Italy
Genoa
Italy
Latina
Italy
Palermo
Italy
Rome
Italy
Turin
Italy
Viterbo
Italy
Aichi
Japan
Akita
Japan
Aomori
Japan
Chiba
Japan
Ehime
Japan
Fukushima
Japan
Fukui
Japan
Fukuoka
Japan
Gifu
Japan
Gunma
Japan
Hokkaido
Japan
Hiroshima
Japan
Hyogo
Japan
Ibaraki
Japan
Ishikawa
Japan
Iwate
Japan
Kagawa
Japan
Early
Late
RR
Summer Temperature

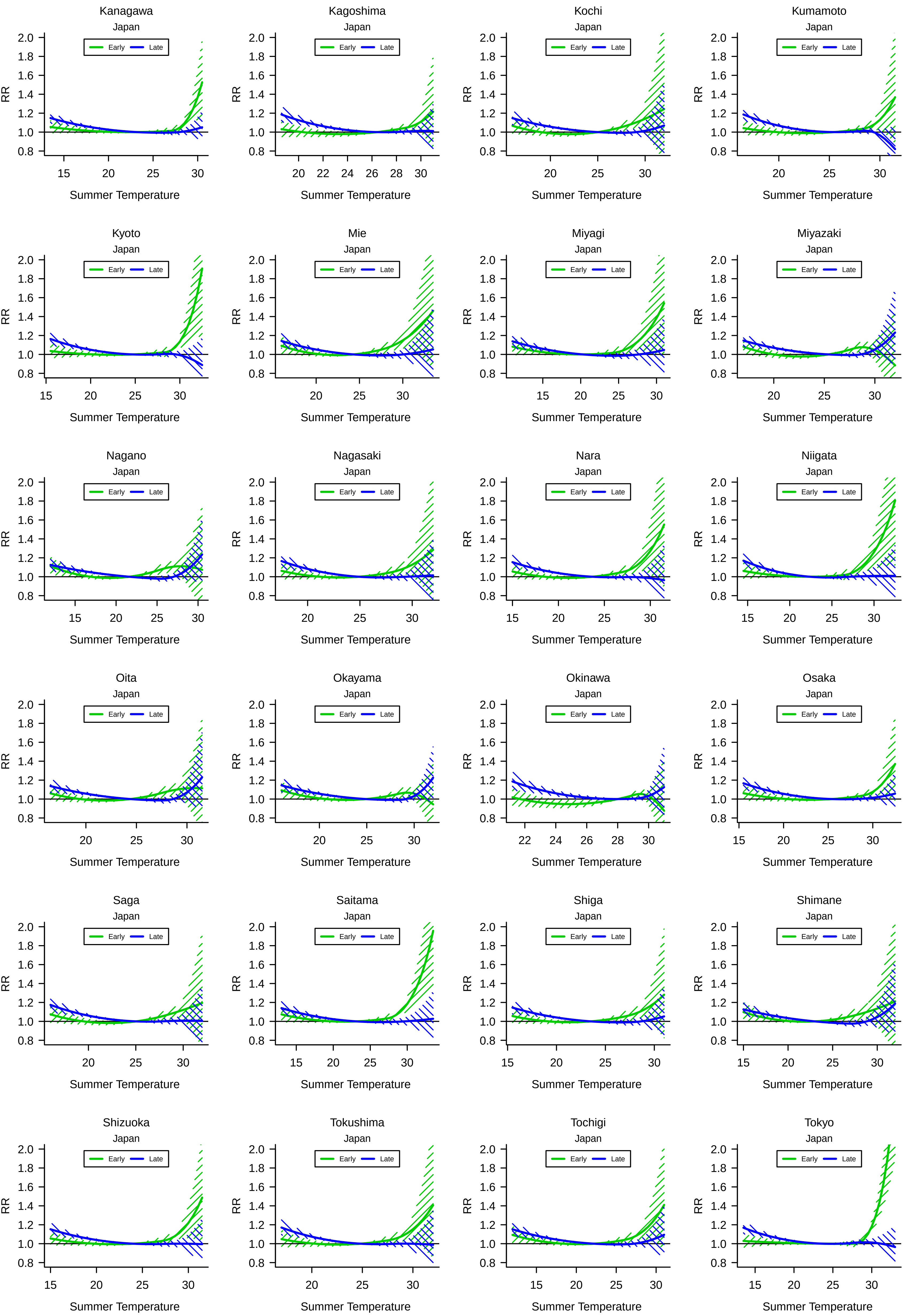
Kanagawa
Japan
Early
Late
RR
2.0 1.8 1.6 1.4 1.2 1.0 0.8
15 20 25 30
Summer Temperature
Kagoshima
Japan
Early
Late
RR
20 22 24 26 28 30
Summer Temperature
Kochi
Japan
Early
Late
RR
20 25 30
Summer Temperature
Kumamoto
Japan
Early
Late
RR
20 25 30
Summer Temperature
Kyoto
Japan
Early
Late
RR
15 20 25 30
Summer Temperature
Mie
Japan
Early
Late
RR
20 25 30
Summer Temperature
Miyagi
Japan
Early
Late
RR
15 20 25 30
Summer Temperature
Miyazaki
Japan
Early
Late
RR
20 25 30
Summer Temperature
Nagano
Japan
Early
Late
RR
15 20 25 30
Summer Temperature
Nagasaki
Japan
Early
Late
RR
20 25 30
Summer Temperature
Nara
Japan
Early
Late
RR
15 20 25 30
Summer Temperature
Niigata
Japan
Early
Late
RR
15 20 25 30
Summer Temperature
Oita
Japan
Early
Late
RR
20 25 30
Summer Temperature
Okayama
Japan
Early
Late
RR
20 25 30
Summer Temperature
Okinawa
Japan
Early
Late
RR
22 24 26 28 30
Summer Temperature
Osaka
Japan
Early
Late
RR
15 20 25 30
Summer Temperature
Saga
Japan
Early
Late
RR
20 25 30
Summer Temperature
Saitama
Japan
Early
Late
RR
15 20 25 30
Summer Temperature
Shiga
Japan
Early
Late
RR
15 20 25 30
Summer Temperature
Shimane
Japan
Early
Late
RR
15 20 25 30
Summer Temperature
Shizuoka
Japan
Early
Late
RR
15 20 25 30
Summer Temperature
Tokushima
Japan
Early
Late
RR
20 25 30
Summer Temperature
Tochigi
Japan
Early
Late
RR
15 20 25 30
Summer Temperature
Tokyo
Japan
Early
Late
RR
15 20 25 30
Summer Temperature

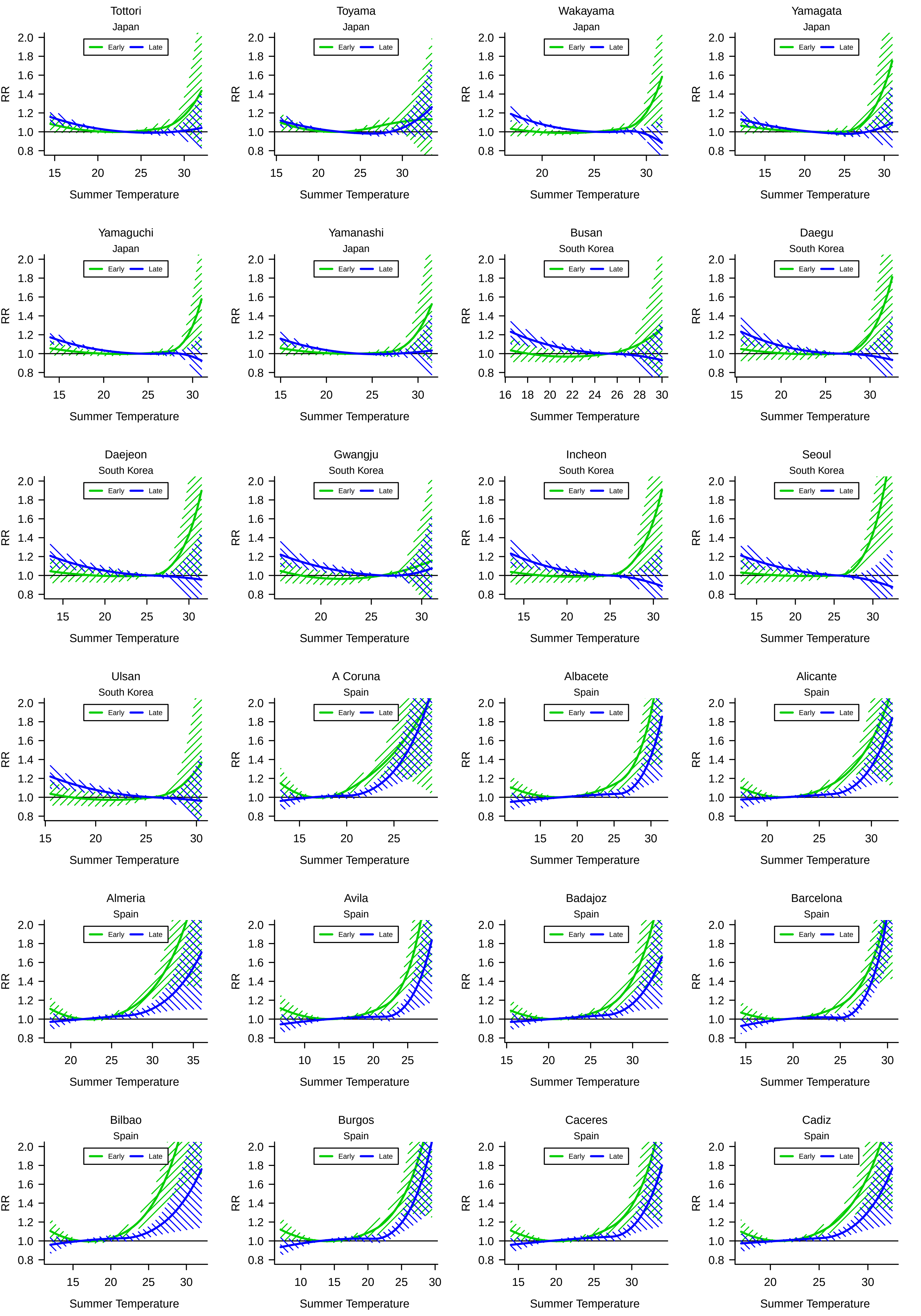
Tottori
Japan
Early Late
RR
2.0 1.8 1.6 1.4 1.2 1.0 0.8
15 20 25 30
Summer Temperature
Toyama
Japan
Early Late
RR
2.0 1.8 1.6 1.4 1.2 1.0 0.8
15 20 25 30
Summer Temperature
Wakayama
Japan
Early Late
RR
2.0 1.8 1.6 1.4 1.2 1.0 0.8
20 25 30
Summer Temperature
Yamagata
Japan
Early Late
RR
2.0 1.8 1.6 1.4 1.2 1.0 0.8
15 20 25 30
Summer Temperature
Yamaguchi
Japan
Early Late
RR
2.0 1.8 1.6 1.4 1.2 1.0 0.8
15 20 25 30
Summer Temperature
Yamanashi
Japan
Early Late
RR
2.0 1.8 1.6 1.4 1.2 1.0 0.8
15 20 25 30
Summer Temperature
Busan
South Korea
Early Late
RR
2.0 1.8 1.6 1.4 1.2 1.0 0.8
16 18 20 22 24 26 28 30
Summer Temperature
Daegu
South Korea
Early Late
RR
2.0 1.8 1.6 1.4 1.2 1.0 0.8
15 20 25 30
Summer Temperature
Daejeon
South Korea
Early Late
RR
2.0 1.8 1.6 1.4 1.2 1.0 0.8
15 20 25 30
Summer Temperature
Gwangju
South Korea
Early Late
RR
2.0 1.8 1.6 1.4 1.2 1.0 0.8
20 25 30
Summer Temperature
Incheon
South Korea
Early Late
RR
2.0 1.8 1.6 1.4 1.2 1.0 0.8
15 20 25 30
Summer Temperature
Seoul
South Korea
Early Late
RR
2.0 1.8 1.6 1.4 1.2 1.0 0.8
15 20 25 30
Summer Temperature
Ulsan
South Korea
Early Late
RR
2.0 1.8 1.6 1.4 1.2 1.0 0.8
15 20 25 30
Summer Temperature
A Coruna
Spain
Early Late
RR
2.0 1.8 1.6 1.4 1.2 1.0 0.8
15 20 25
Summer Temperature
Albacete
Spain
Early Late
RR
2.0 1.8 1.6 1.4 1.2 1.0 0.8
15 20 25 30
Summer Temperature
Alicante
Spain
Early Late
RR
2.0 1.8 1.6 1.4 1.2 1.0 0.8
20 25 30
Summer Temperature
Almeria
Spain
Early Late
RR
2.0 1.8 1.6 1.4 1.2 1.0 0.8
20 25 30 35
Summer Temperature
Avila
Spain
Early Late
RR
2.0 1.8 1.6 1.4 1.2 1.0 0.8
10 15 20 25
Summer Temperature
Badajoz
Spain
Early Late
RR
2.0 1.8 1.6 1.4 1.2 1.0 0.8
15 20 25 30
Summer Temperature
Barcelona
Spain
Early Late
RR
2.0 1.8 1.6 1.4 1.2 1.0 0.8
15 20 25 30
Summer Temperature
Bilbao
Spain
Early Late
RR
2.0 1.8 1.6 1.4 1.2 1.0 0.8
15 20 25 30
Summer Temperature
Burgos
Spain
Early Late
RR
2.0 1.8 1.6 1.4 1.2 1.0 0.8
10 15 20 25 30
Summer Temperature
Caceres
Spain
Early Late
RR
2.0 1.8 1.6 1.4 1.2 1.0 0.8
15 20 25 30
Summer Temperature
Cadiz
Spain
Early Late
RR
2.0 1.8 1.6 1.4 1.2 1.0 0.8
20 25 30
Summer Temperature

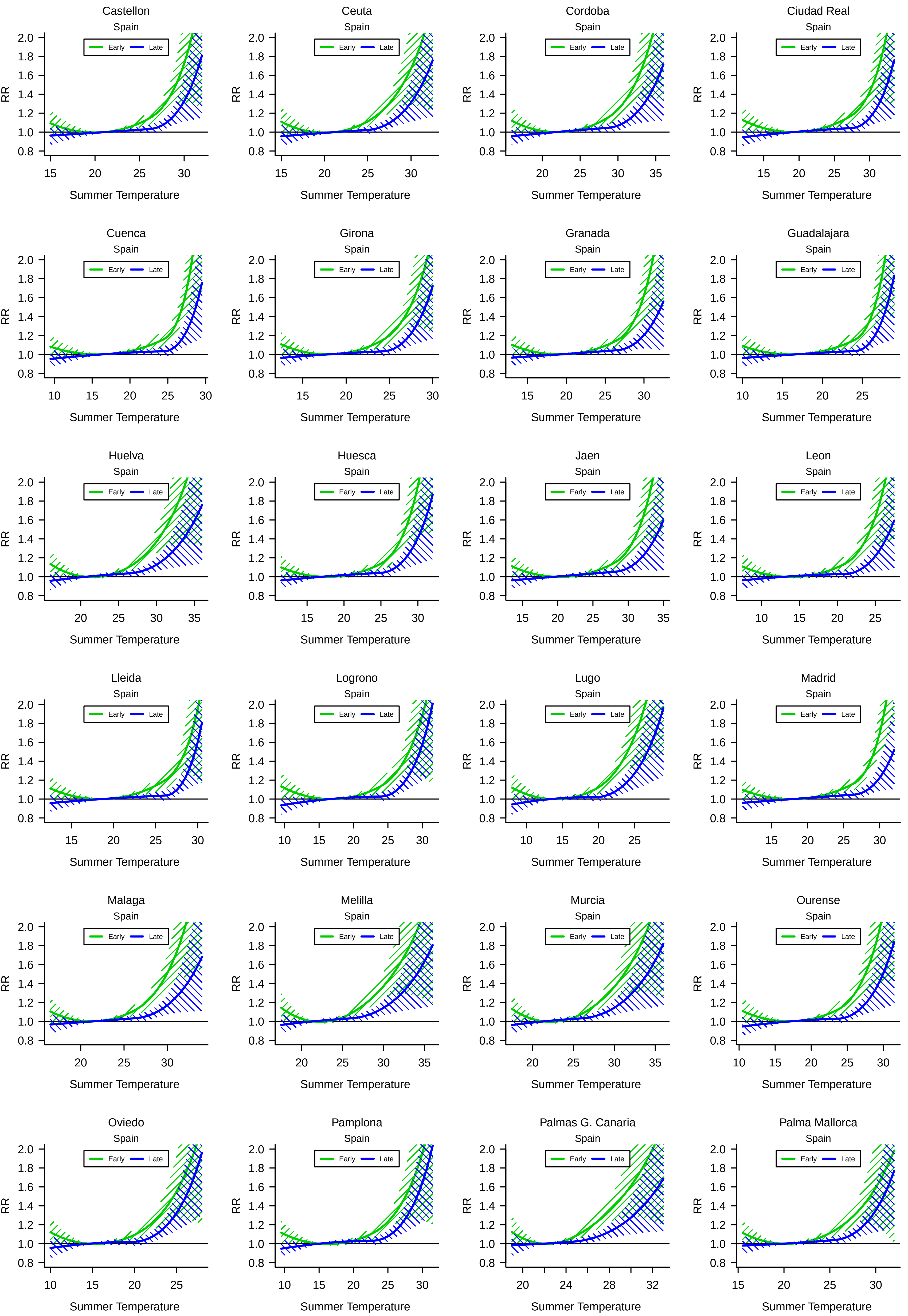
Castellon
Spain
Ceuta
Spain
Cordoba
Spain
Ciudad Real
Spain
Cuenca
Spain
Girona
Spain
Granada
Spain
Guadalajara
Spain
Huelva
Spain
Huesca
Spain
Jaen
Spain
Leon
Spain
Lleida
Spain
Logrono
Spain
Lugo
Spain
Madrid
Spain
Malaga
Spain
Melilla
Spain
Murcia
Spain
Ourense
Spain
Oviedo
Spain
Pamplona
Spain
Palmas G. Canaria
Spain
Palma Mallorca
Spain
Early
Late
RR
Summer Temperature

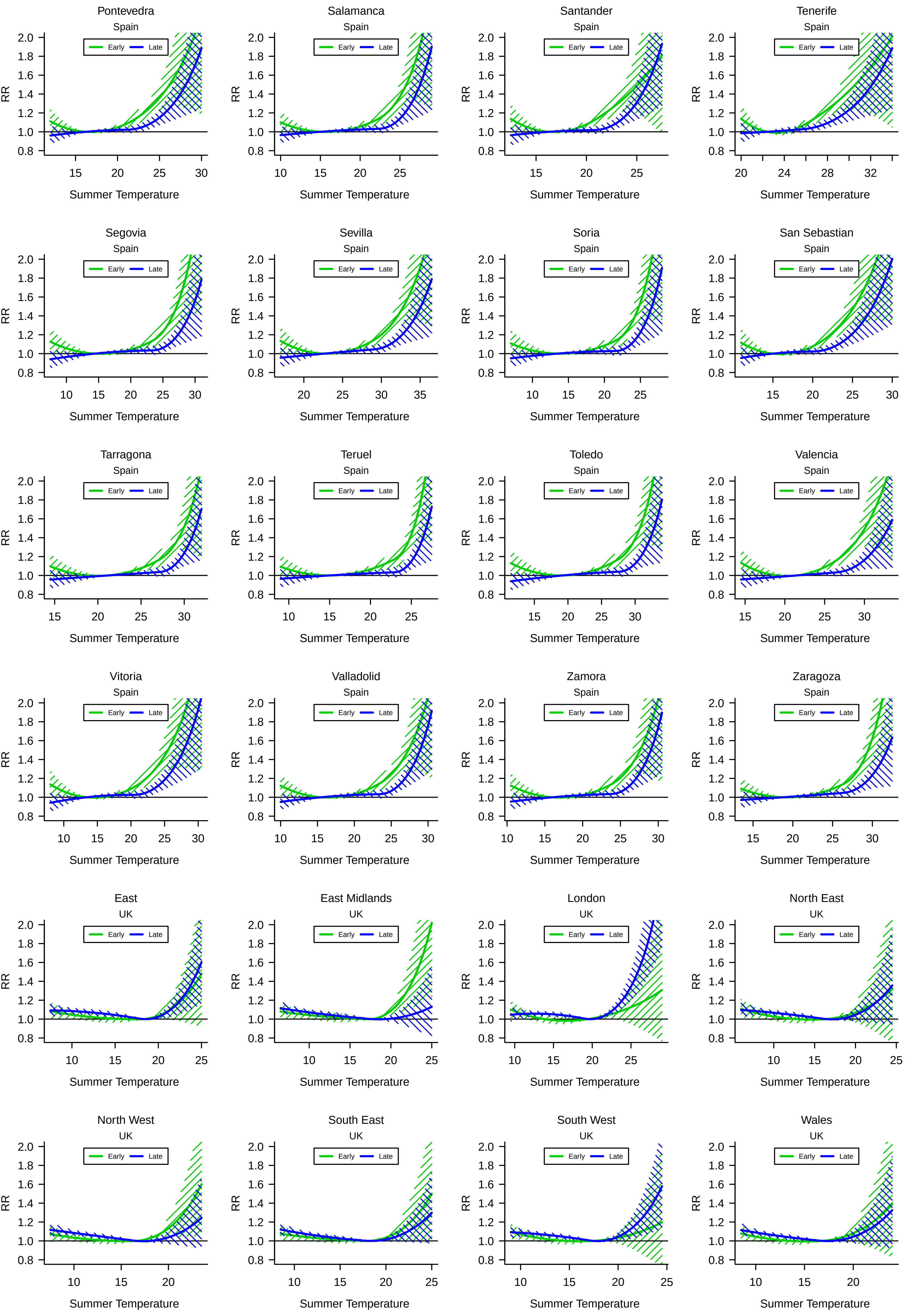
Pontevedra
Spain
Salamanca
Spain
Santander
Spain
Tenerife
Spain
Segovia
Spain
Sevilla
Spain
Soria
Spain
San Sebastian
Spain
Tarragona
Spain
Teruel
Spain
Toledo
Spain
Valencia
Spain
Vitoria
Spain
Valladolid
Spain
Zamora
Spain
Zaragoza
Spain
East
UK
East Midlands
UK
London
UK
North East
UK
North West
UK
South East
UK
South West
UK
Wales
UK
Early
Late
RR
Summer Temperature

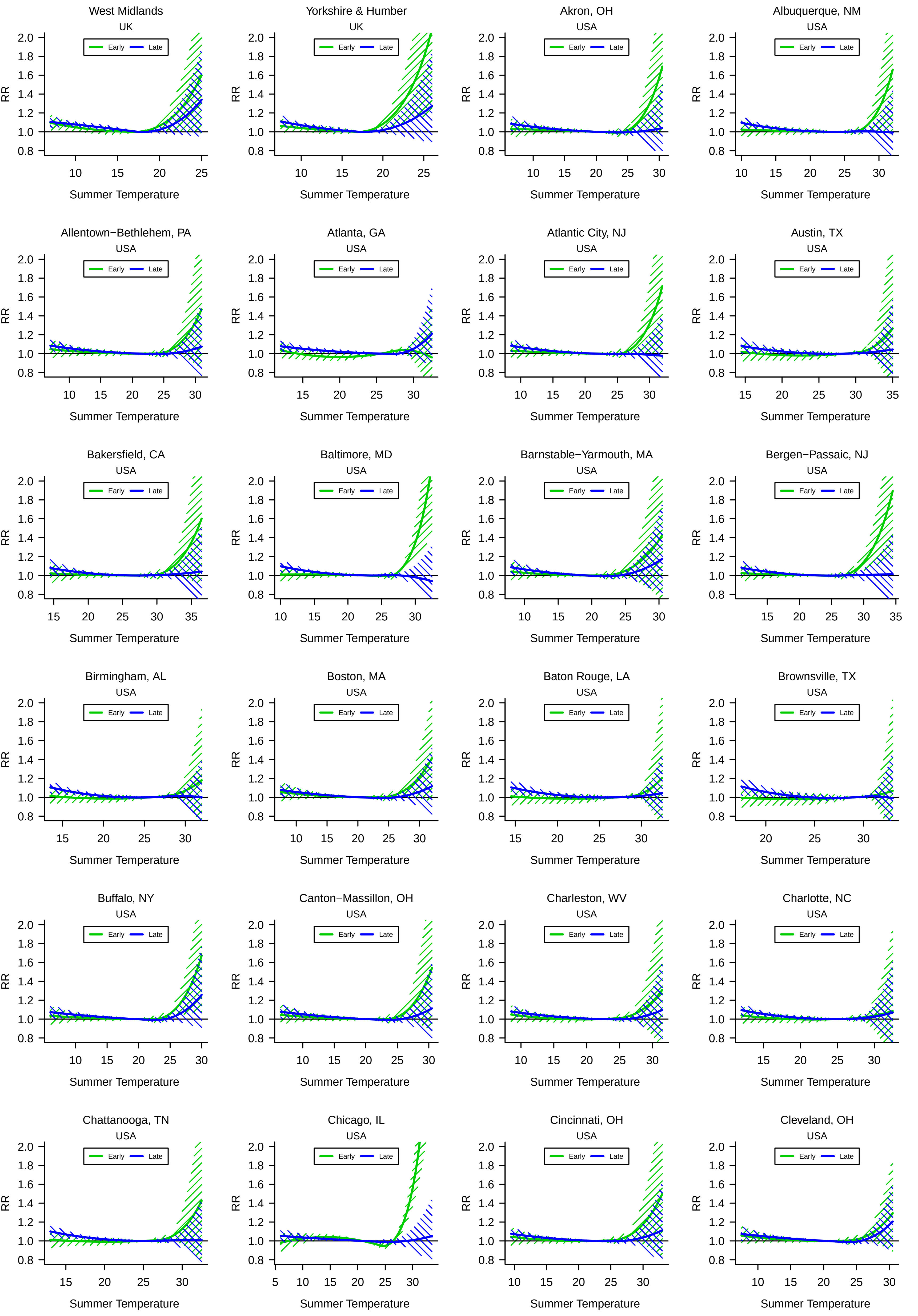
West Midlands
UK
Yorkshire & Humber
UK
Akron, OH
USA
Albuquerque, NM
USA
Allentown−Bethlehem, PA
USA
Atlanta, GA
USA
Atlantic City, NJ
USA
Austin, TX
USA
Bakersfield, CA
USA
Baltimore, MD
USA
Barnstable−Yarmouth, MA
USA
Bergen−Passaic, NJ
USA
Birmingham, AL
USA
Boston, MA
USA
Baton Rouge, LA
USA
Brownsville, TX
USA
Buffalo, NY
USA
Canton−Massillon, OH
USA
Charleston, WV
USA
Charlotte, NC
USA
Chattanooga, TN
USA
Chicago, IL
USA
Cincinnati, OH
USA
Cleveland, OH
USA
Early
Late
RR
Summer Temperature

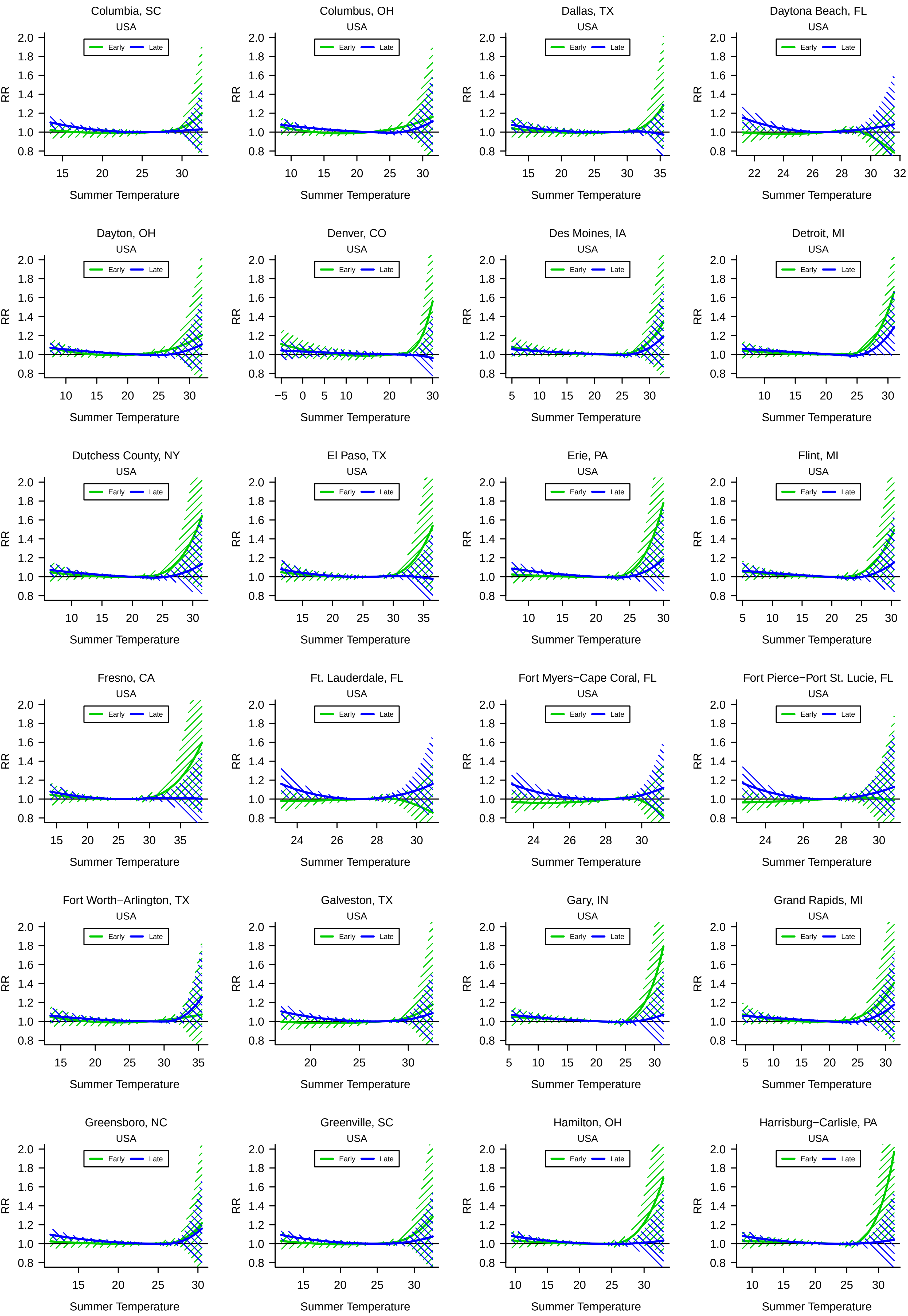
Columbia, SC
USA
Columbus, OH
USA
Dallas, TX
USA
Daytona Beach, FL
USA
Dayton, OH
USA
Denver, CO
USA
Des Moines, IA
USA
Detroit, MI
USA
Dutchess County, NY
USA
El Paso, TX
USA
Erie, PA
USA
Flint, MI
USA
Fresno, CA
USA
Ft. Lauderdale, FL
USA
Fort Myers−Cape Coral, FL
USA
Fort Pierce−Port St. Lucie, FL
USA
Fort Worth−Arlington, TX
USA
Galveston, TX
USA
Gary, IN
USA
Grand Rapids, MI
USA
Greensboro, NC
USA
Greenville, SC
USA
Hamilton, OH
USA
Harrisburg−Carlisle, PA
USA
Early
Late
RR
Summer Temperature

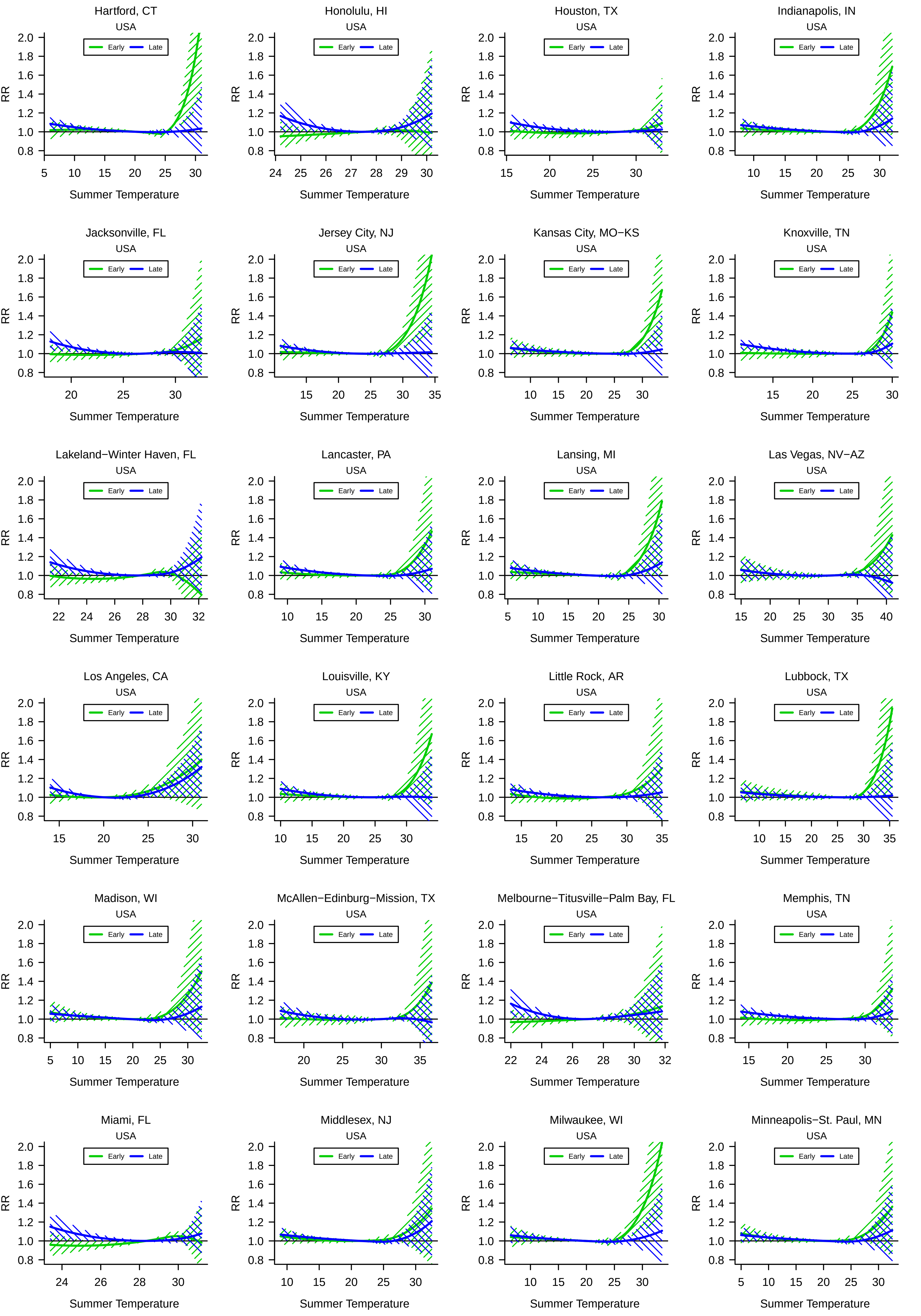
Hartford, CT
USA
Honolulu, HI
USA
Houston, TX
USA
Indianapolis, IN
USA
Jacksonville, FL
USA
Jersey City, NJ
USA
Kansas City, MO−KS
USA
Knoxville, TN
USA
Lakeland−Winter Haven, FL
USA
Lancaster, PA
USA
Lansing, MI
USA
Las Vegas, NV−AZ
USA
Los Angeles, CA
USA
Louisville, KY
USA
Little Rock, AR
USA
Lubbock, TX
USA
Madison, WI
USA
McAllen−Edinburg−Mission, TX
USA
Melbourne−Titusville−Palm Bay, FL
USA
Memphis, TN
USA
Miami, FL
USA
Middlesex, NJ
USA
Milwaukee, WI
USA
Minneapolis−St. Paul, MN
USA
Early
Late
RR
Summer Temperature

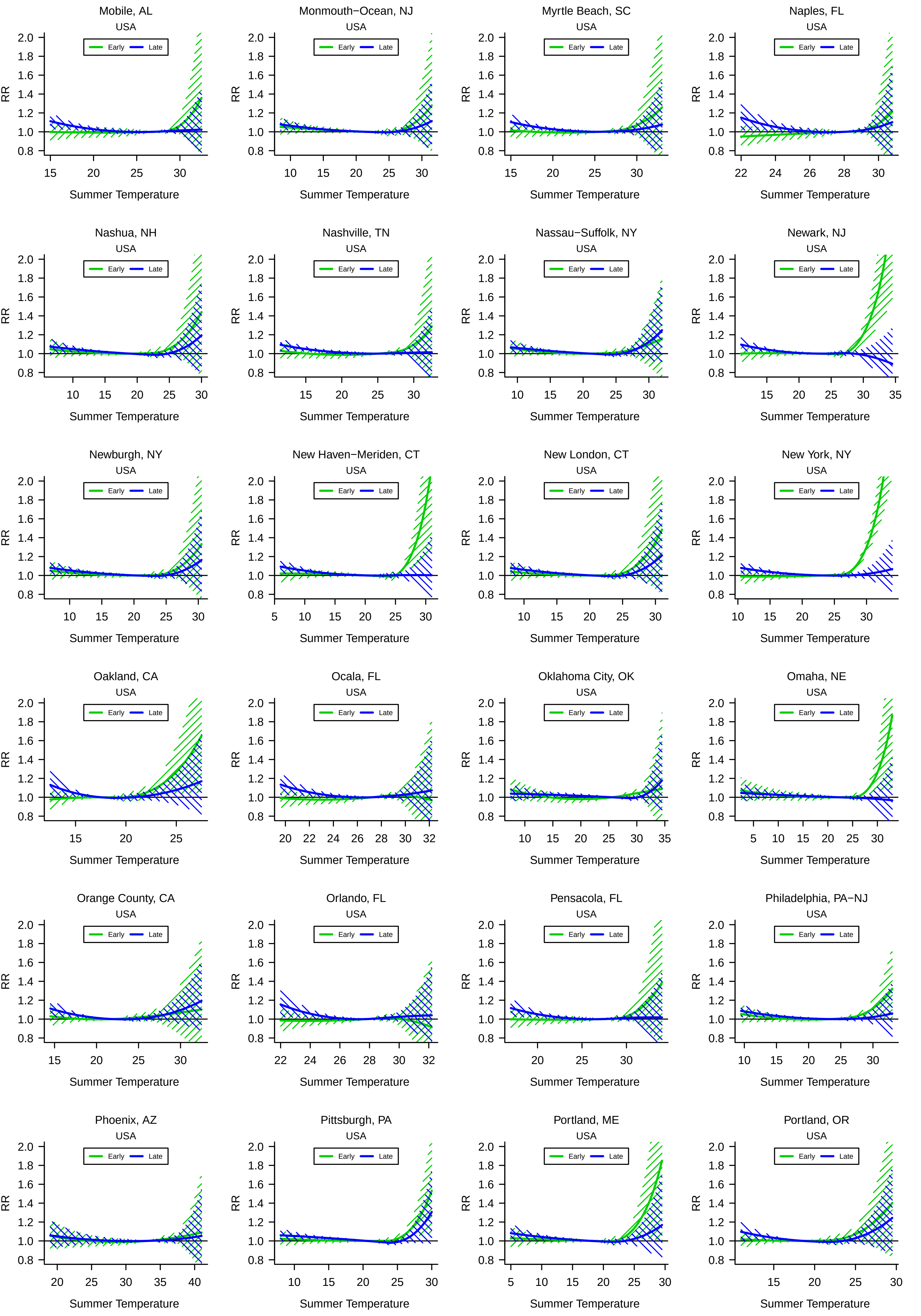
Mobile, AL
USA
Monmouth−Ocean, NJ
USA
Myrtle Beach, SC
USA
Naples, FL
USA
Nashua, NH
USA
Nashville, TN
USA
Nassau−Suffolk, NY
USA
Newark, NJ
USA
Newburgh, NY
USA
New Haven−Meriden, CT
USA
New London, CT
USA
New York, NY
USA
Oakland, CA
USA
Ocala, FL
USA
Oklahoma City, OK
USA
Omaha, NE
USA
Orange County, CA
USA
Orlando, FL
USA
Pensacola, FL
USA
Philadelphia, PA−NJ
USA
Phoenix, AZ
USA
Pittsburgh, PA
USA
Portland, ME
USA
Portland, OR
USA
Early
Late
RR
Summer Temperature

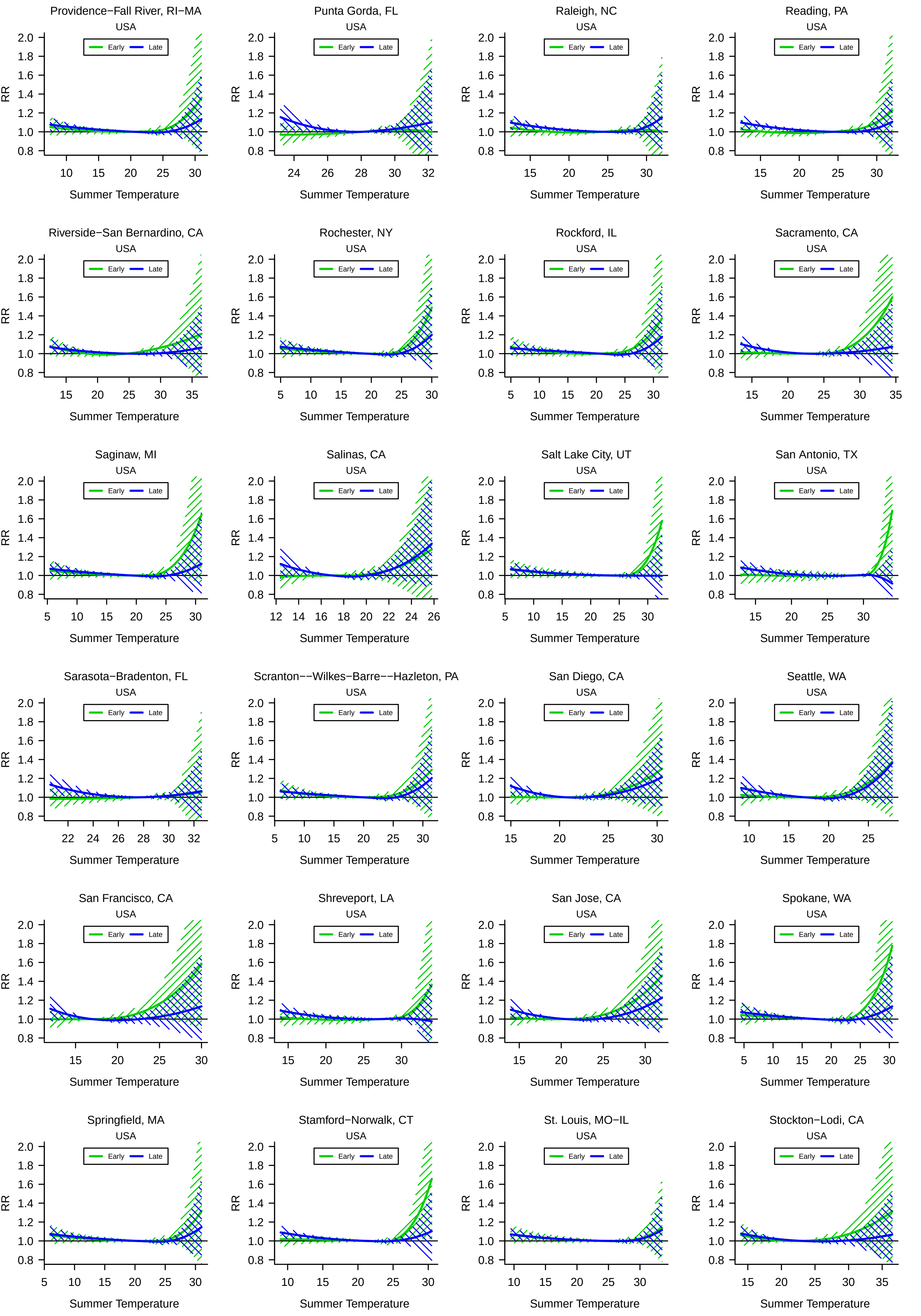
Providence−Fall River, RI−MA
USA
Punta Gorda, FL
USA
Raleigh, NC
USA
Reading, PA
USA
Riverside−San Bernardino, CA
USA
Rochester, NY
USA
Rockford, IL
USA
Sacramento, CA
USA
Saginaw, MI
USA
Salinas, CA
USA
Salt Lake City, UT
USA
San Antonio, TX
USA
Sarasota−Bradenton, FL
USA
Scranton−−Wilkes−Barre−−Hazleton, PA
USA
San Diego, CA
USA
Seattle, WA
USA
San Francisco, CA
USA
Shreveport, LA
USA
San Jose, CA
USA
Spokane, WA
USA
Springfield, MA
USA
Stamford−Norwalk, CT
USA
St. Louis, MO−IL
USA
Stockton−Lodi, CA
USA
Early
Late
RR
Summer Temperature

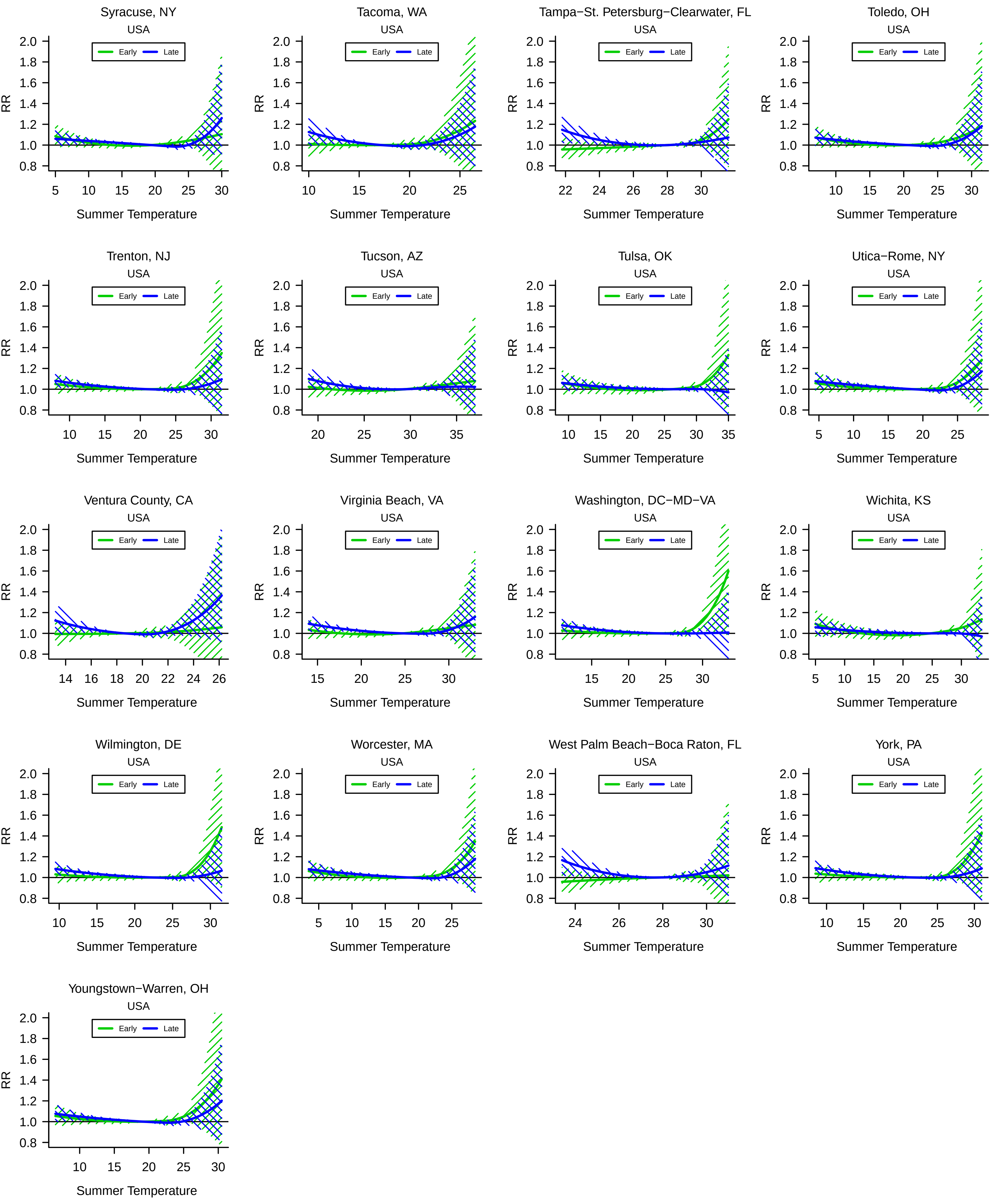
Syracuse, NY
USA
Tacoma, WA
USA
Tampa−St. Petersburg−Clearwater, FL
USA
Toledo, OH
USA
Trenton, NJ
USA
Tucson, AZ
USA
Tulsa, OK
USA
Utica−Rome, NY
USA
Ventura County, CA
USA
Virginia Beach, VA
USA
Washington, DC−MD−VA
USA
Wichita, KS
USA
Wilmington, DE
USA
Worcester, MA
USA
West Palm Beach−Boca Raton, FL
USA
York, PA
USA
Youngstown−Warren, OH
USA
Early
Late
RR
Summer Temperature

Web Figure 4. Effect modification of day of season on the overalll cumulative exposure-response relationships between heat and mortality in 9 countries, expressed as late/early ratio of RRs (corresponding to the mid-point of the last and first summer months, respectively), with 95% confidence intervals. The curves are represented on a relative scale of summer temperature percentiles, using country-specific distributions. The vertical lines represent the average minimum mortality percentile (dotted) and the 90th and 99th percentiles of the temperature distribution (dashed). Note that the *y*-axis is scaled to the country-specific range.

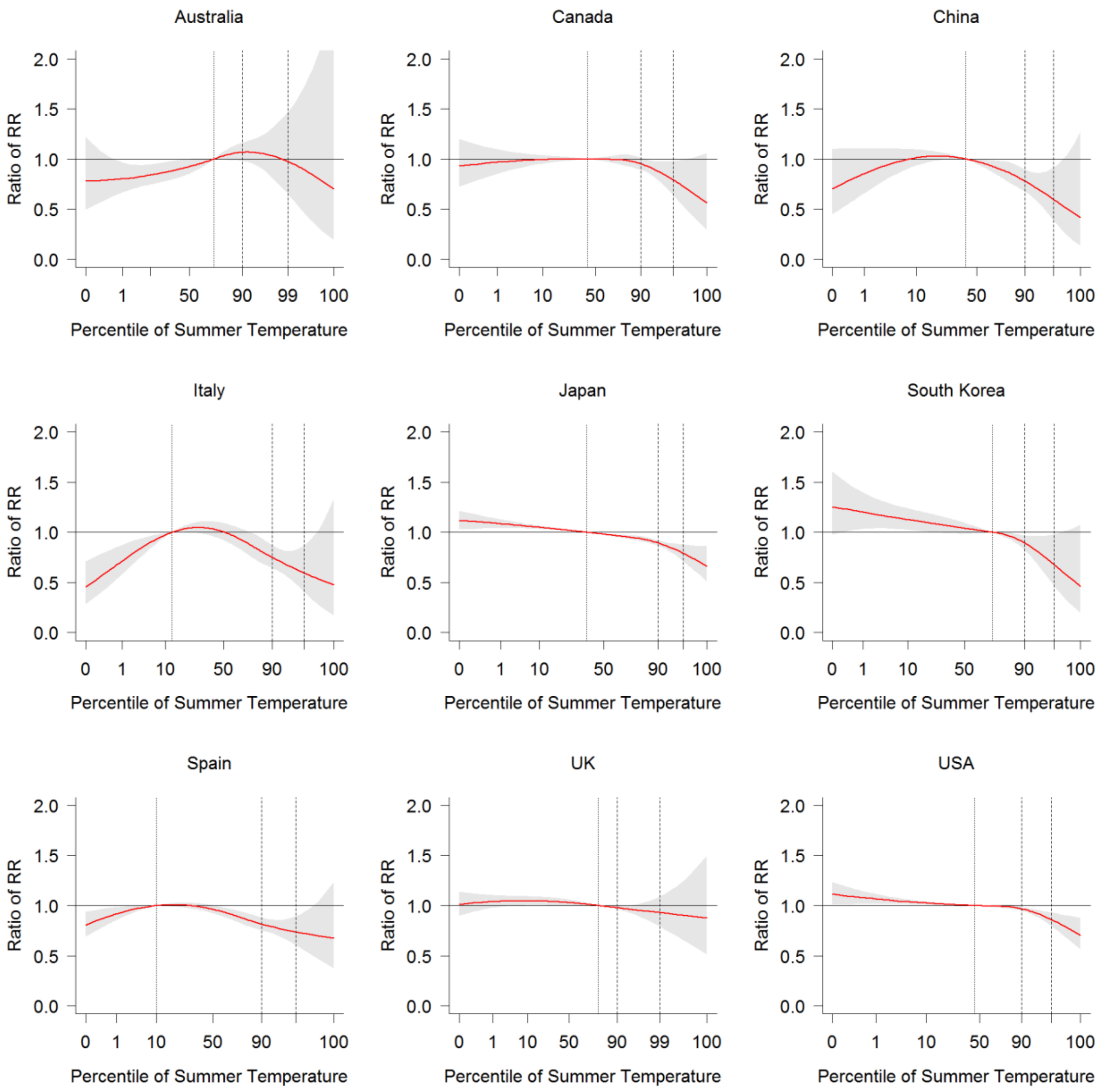

Web Figure 5. Replication of Figure 3 in the manuscript, including colours and confidence intervals as shaded areas.

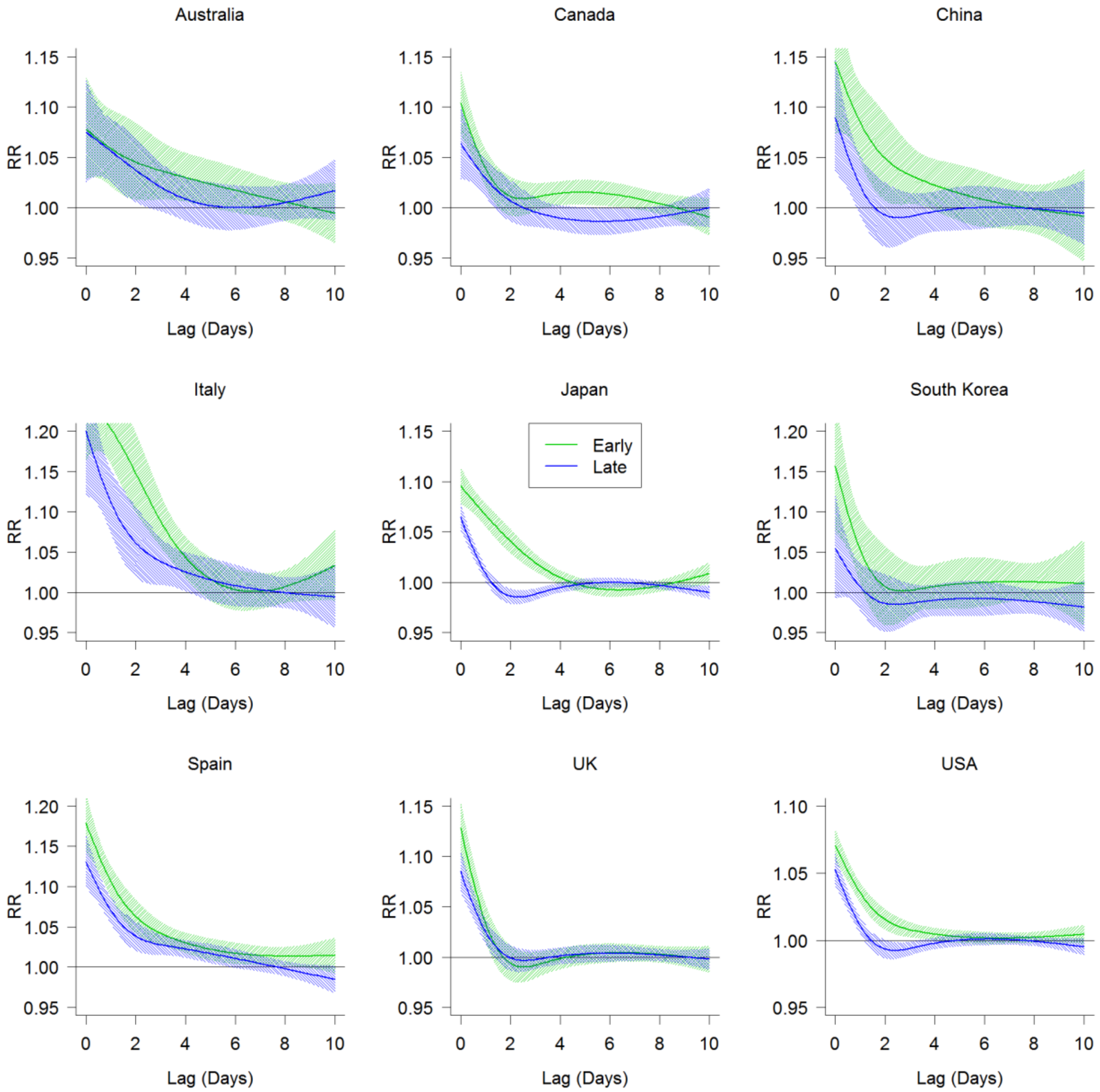

Web Figure 6. Replication of Figure 3 in the manuscript for the average across the summer, with confidence intervals as grey areas.

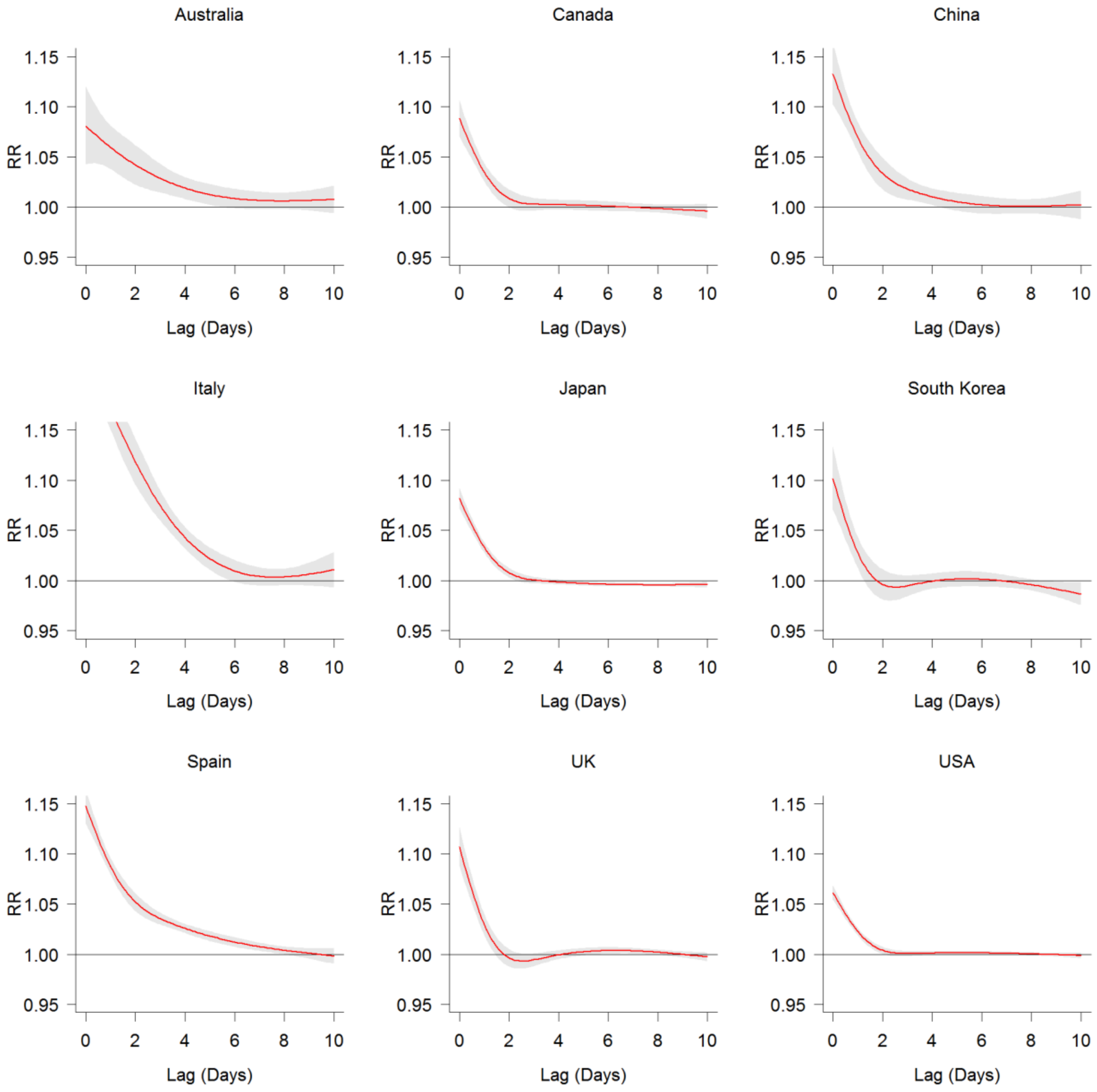

Web Figure 7. Replication of Figure 3 in the manuscript using a quadratic B-spline function with 2 df (no knots) as the exposure-response function.

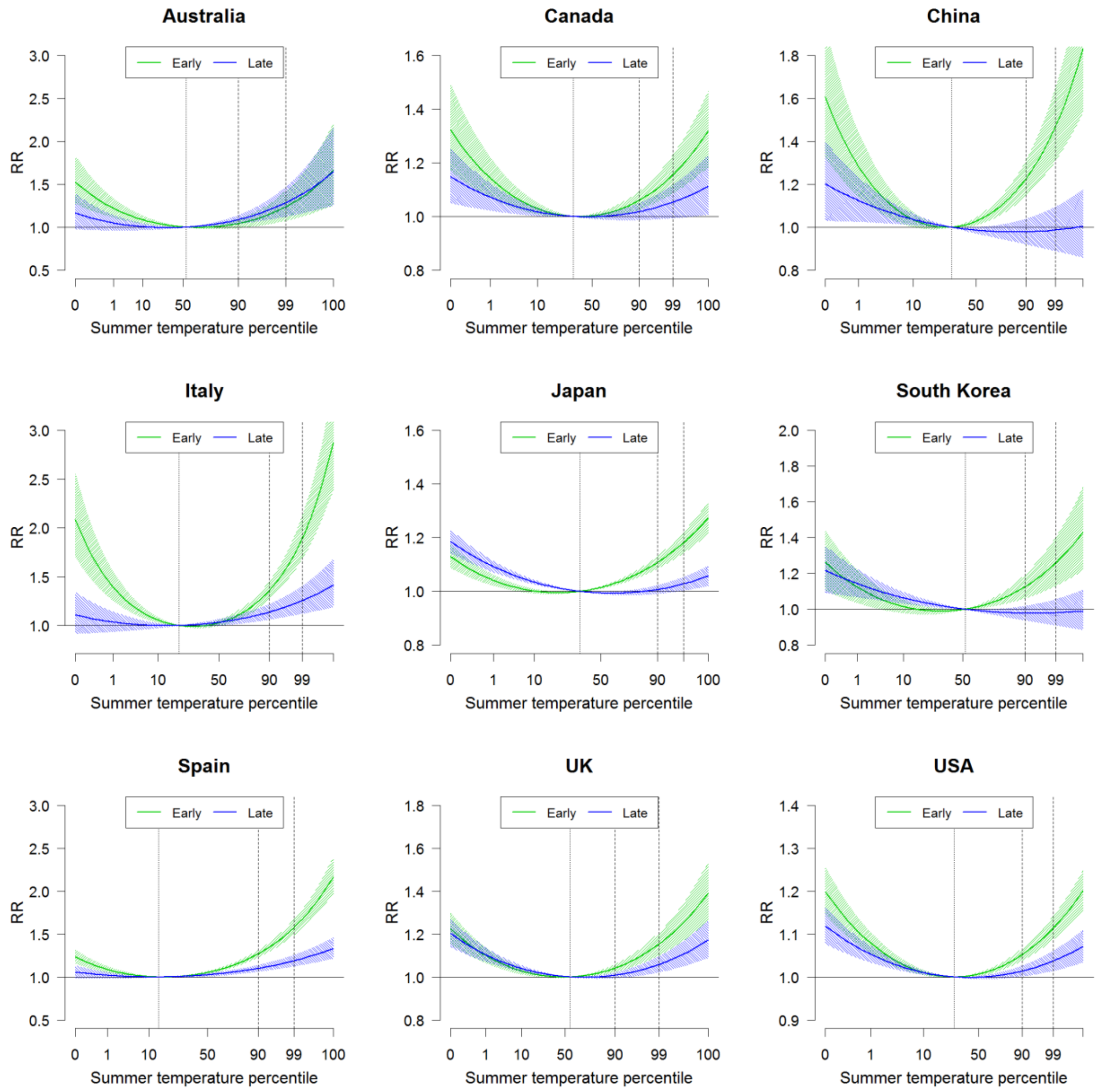

Web Figure 8. Replication of Figure 3 in the manuscript using a quadratic B-spline function with 4 df (knots at the 50th and 90th percentile of summer temperature distribution) as the exposure-response function.

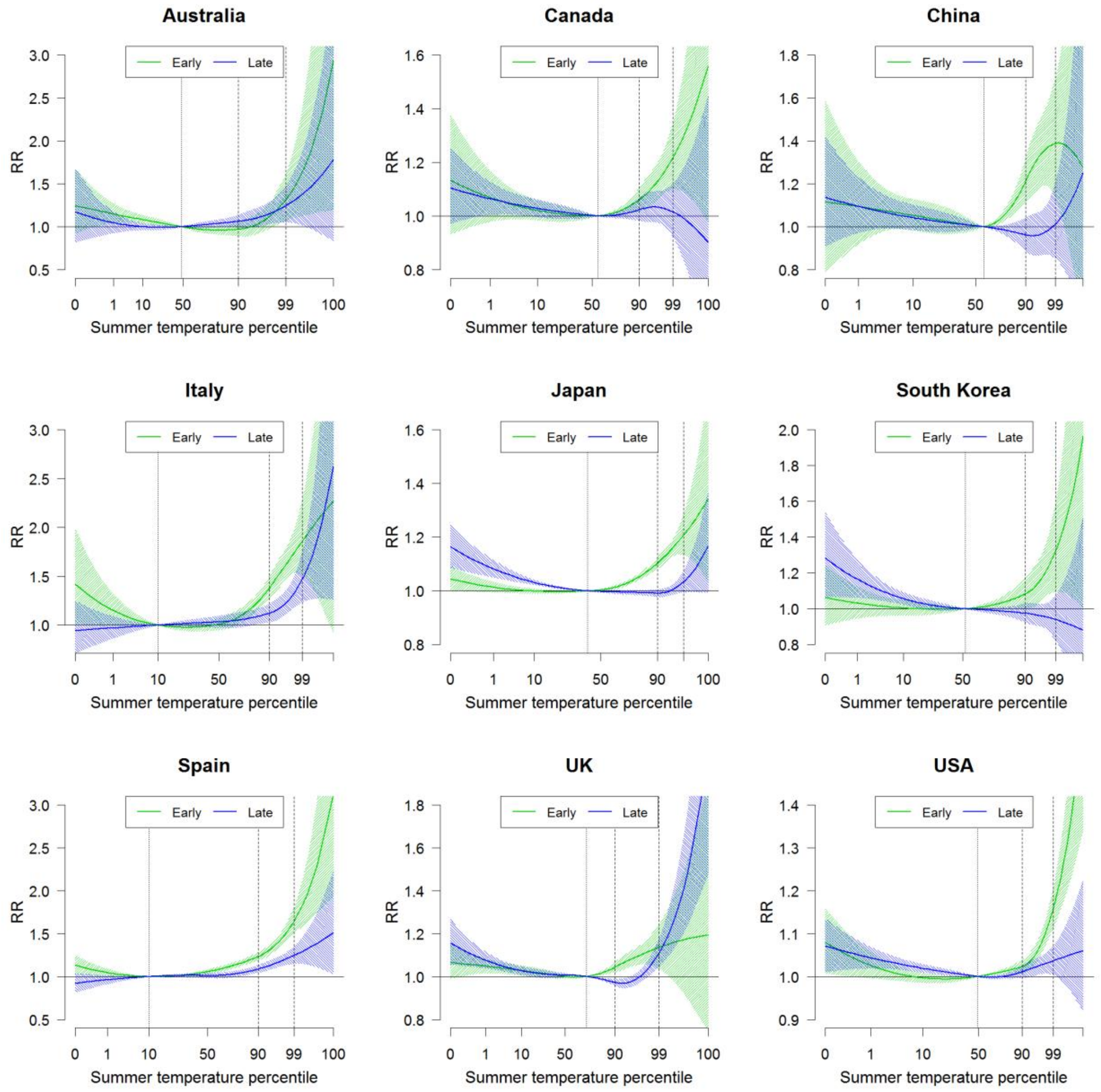

Web Figure 9. Replication of Figure 3 in the manuscript (excluding confidence intervals), with and without control for relative humidity, modelled using a natural cubic B-spline function with 3 df of the average over lag 0-1. The analysis is performed in the subset of 223 locations including at least 80% of daily measurement of relative humidity.

[a]The curves are represented on a relative scale of summer temperature percentiles, using country-specific distributions.

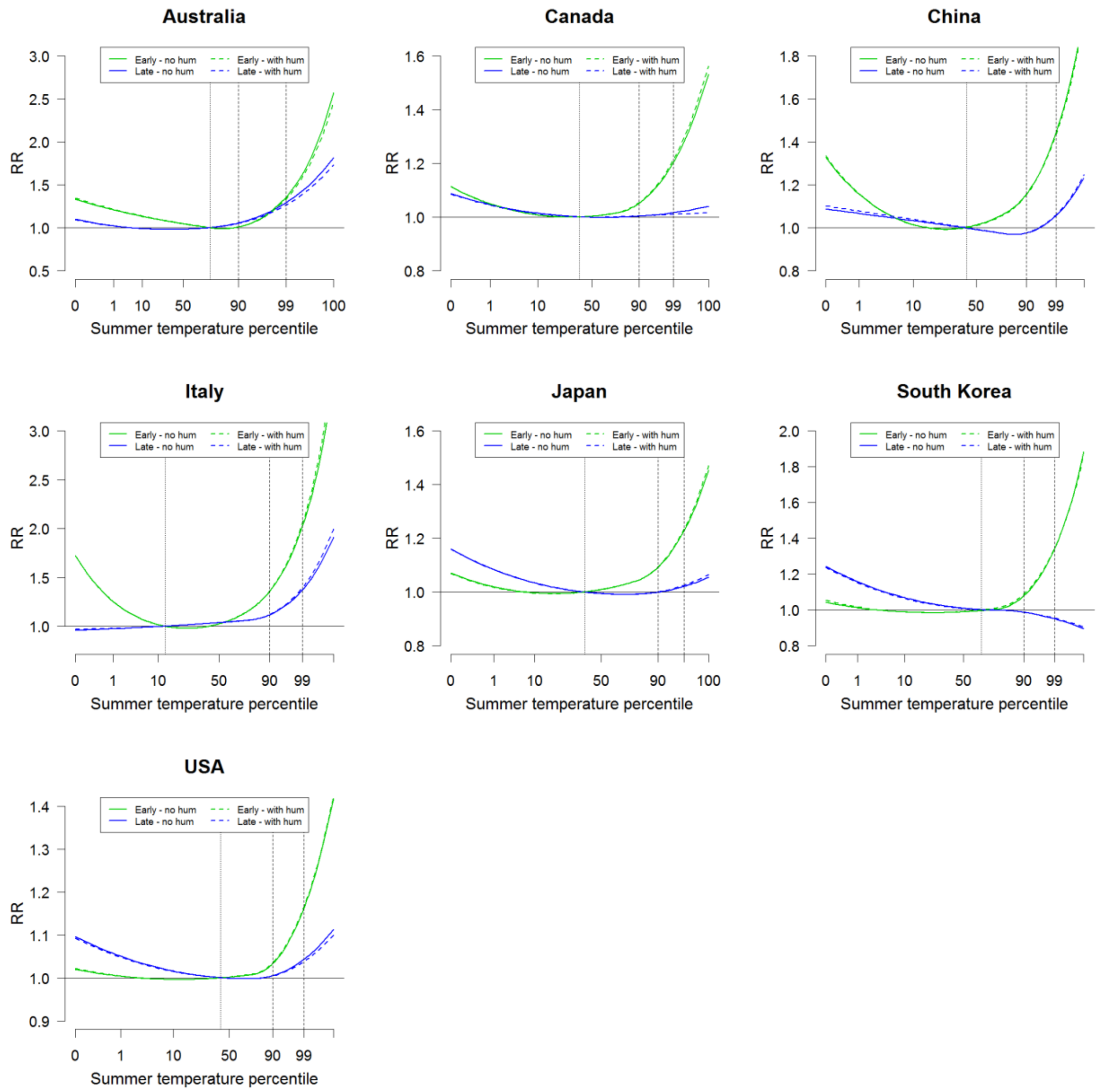

Web Figure 10. Replication of Figure 3 in the manuscript (excluding confidence intervals), with and without control for diurnal temperature range (DTR). The analysis is performed in the subset of 273 locations including at least 80% of daily measurement of maximum and minimum daily temperature.

[a]The curves are represented on a relative scale of summer temperature percentiles, using country-specific distributions.

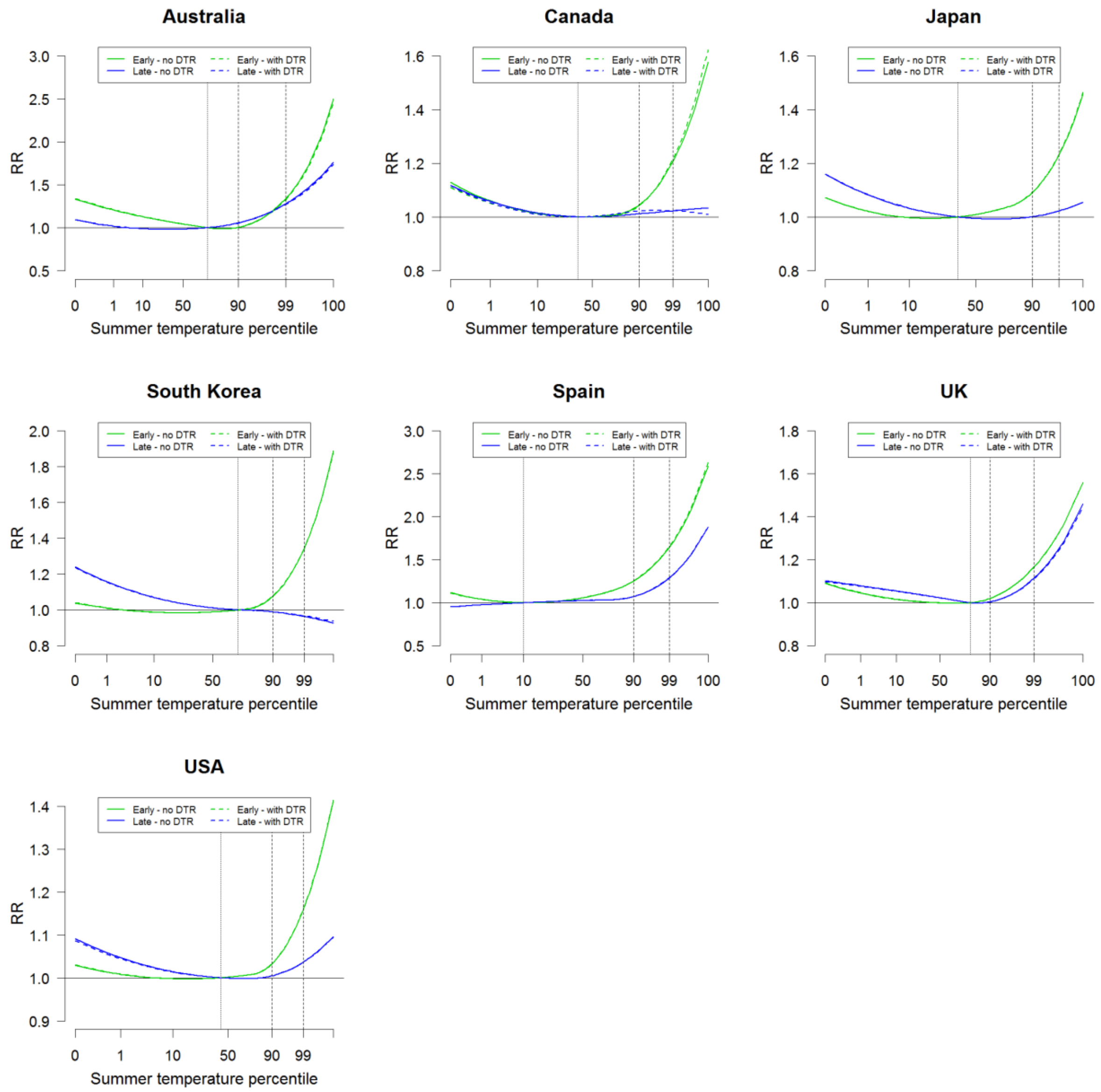

References

1. Tong S, Wang XY, Guo Y. Assessing the short-term effects of heatwaves on mortality and morbidity in Brisbane, Australia: comparison of case-crossover and time series analyses. *PloS One* 2012;7(5):e37500.
2. Tong S, Wang XY, Yu W, et al. The impact of heatwaves on mortality in Australia: a multicity study. *BMJ Open* 2014;4(2):e003579.
3. Gasparrini A, Guo Y, Hashizume M, et al. Mortality risk attributable to high and low ambient temperature: a multicountry observational study. *The Lancet* 2015;386(9991):369-375.
4. Gasparrini A, Guo Y, Hashizume M, et al. Temporal variation in heat-mortality associations: a multi-country study. *Environmental Health Perspectives* 2015; May 1 [Epub ahead of print]. DOI: 10.1289/ehp.1409070.
5. Kaplan GG, Tanyingoh D, Dixon E, et al. Ambient ozone concentrations and the risk of perforated and nonperforated appendicitis: a multicity case-crossover study. *Environmental Health Perspectives* 2013;121(8):939-943.
6. Martin SL, Cakmak S, Hebbern CA, et al. Climate change and future temperature-related mortality in 15 Canadian cities. *International Journal of Biometeorology* 2012;56(4):605-619.
7. Michelozzi P, de'Donato FK, Bargagli AM, et al. Surveillance of summer mortality and preparedness to reduce the health impact of heat waves in Italy. *International Journal of Environmental Research and Public Health* 2010;7(5):2256-2273.
8. Schifano P, Leone M, De Sario M, et al. Changes in the effects of heat on mortality among the elderly from 1998--2010: results from a multicenter time series study in Italy. *Environ Health* 2012;11(1):58.
9. Tobias A, Armstrong B, Zuza I, et al. Mortality on extreme heat days using official thresholds in Spain: a multi-city time series analysis. *BMC Public Health* 2012;12(1):133.
10. Armstrong BG, Chalabi Z, Fenn B, et al. The association of mortality with high temperatures in a temperate climate: England and Wales. *Journal of Epidemiology and Community Health* 2011;65(4):340-345.
11. Gasparrini A, Armstrong B, Kovats S, et al. The effect of high temperatures on cause-specific mortality in England and Wales. *Occupational and Environmental Medicine* 2012;69(1):56-61.
12. Zanobetti A, O'Neill MS, Gronlund CJ, et al. Susceptibility to mortality in weather extremes: effect modification by personal and small-area characteristics. *Epidemiology* 2013;24(6):809-819.
13. Zanobetti A, Schwartz J. The effect of fine and coarse particulate air pollution on mortality: a national analysis. *Environmental Health Perspectives* 2009;117(6):898-903.
14. R Core Team. R: A Language and Environment for Statistical Computing. Vienna, Austria: R Foundation for Statistical Computing, 2015.
15. Gasparrini A. Distributed lag linear and non-linear models in R: the package dlnm. *Journal of Statistical Software* 2011;43(8):1-20.
16. Gasparrini A, Armstrong B, Kenward MG. Multivariate meta-analysis for non-linear and other multi-parameter associations. *Statistics in Medicine* 2012;31(29):3821-3839.
